# Common Inflammatory Pathways Between Periodontal Disease and Multiple Sclerosis: A Systematic Review

**DOI:** 10.3390/diseases14080268

**Published:** 2026-07-24

**Authors:** Vasile Calin Arcas, Iulian Roman-Filip, Doru Florian Cornel Moga, Adriana Saceleanu, Anca Maria Fratila, Lucia Nicola Fratila, Corina Roman-Filip

**Affiliations:** 1 Faculty of Medicine, Lucian Blaga University of Sibiu, 550169 Sibiu, Romania; calin.arcas@ulbsibiu.ro (V.C.A.); adriana.saceleanu@ulbsibiu.ro (A.S.); anca.fratila@ulbsibiu.ro (A.M.F.); lucianicola.fratila@ulbsibiu.ro (L.N.F.); corina.roman@ulbsibiu.ro (C.R.-F.); 2 Department of Neurology, “George Emil Palade” University of Medicine, Pharmacy, Sciences and Technology, 540136 Targu Mures, Romania; 3 Military Clinical Emergency Hospital of Sibiu, 550024 Sibiu, Romania; 4 Sibiu County Emergency Clinical Hospital, 550245 Sibiu, Romania

**Keywords:** multiple sclerosis, periodontal diseases, inflammation, cytokines, oral microbiome, neuroinflammation

## Abstract

**Background:** Multiple sclerosis and periodontal disease are chronic inflammatory conditions that may share immune-mediated mechanisms, including cytokine activation, oral dysbiosis, oxidative stress, and systemic inflammatory burden. This systematic review aimed to synthesize recent evidence on common inflammatory pathways linking periodontal disease and multiple sclerosis. **Methods:** The review was conducted according to PRISMA 2020 guidelines. PubMed/MEDLINE, Cochrane Library, and Scopus were searched for English-language studies published between June 2020 and June 2026. Eligible studies addressed multiple sclerosis, periodontal disease, oral microbiome alterations, systemic inflammation, or neuroinflammatory outcomes. Study selection and data extraction were performed independently by three reviewers. Risk of bias was assessed using AMSTAR 2, the Newcastle–Ottawa Scale, and the Joanna Briggs Institute checklist, according to study design. **Results:** Seventeen studies were included in the qualitative synthesis. The main shared mechanisms were cytokine-mediated inflammation involving TNF-α, IL-1β, IL-6, and IL-17; NF-κB signaling; Th17/Treg imbalance; blood–brain barrier disruption; oxidative stress; matrix metalloproteinase activity; complement activation; and oral–gut–brain axis dysregulation. The evidence suggests that periodontal inflammation may contribute to systemic immune activation and may amplify neuroinflammatory processes in multiple sclerosis. **Conclusions:** Current evidence supports a biologically possible association between periodontal disease and multiple sclerosis through shared inflammatory and microbial pathways. However, causality remains unproven, and further longitudinal and interventional studies are needed.

## 1. Introduction

Multiple sclerosis and periodontal disease are distinct chronic conditions affecting different anatomical systems [1,2]: multiple sclerosis primarily involves the central nervous system, where immune-mediated inflammation contributes to demyelination, axonal injury, and neurological dysfunction [3], whereas periodontal disease affects the tooth-supporting tissues, leading to gingival inflammation, connective tissue destruction, attachment loss, and alveolar bone resorption [1,2,4]. Although they do not share the same primary cause, these diseases may interact indirectly through overlapping inflammatory mechanisms. Periodontitis should not be presented as a proven cause of multiple sclerosis, nor should multiple sclerosis be considered a direct cause of periodontal disease. Rather, their potential relationship may involve systemic inflammatory burden, immune dysregulation, oral dysbiosis, cytokine activation, oxidative stress, and possible effects on blood–brain barrier integrity [2]. Therefore, periodontal disease may be relevant in patients with multiple sclerosis as a potential inflammatory comorbidity, while the association between the two conditions should be interpreted as a convergence of shared immunoinflammatory pathways rather than a direct causal relationship [5].

Multiple sclerosis is a chronic immune-mediated inflammatory disease of the central nervous system in which immune dysregulation plays a central role in lesion formation and disease progression [3,6]. Its pathogenesis involves the activation of autoreactive T cells, B-cell responses, macrophage infiltration, and microglial activation, leading to sustained neuroinflammation [3,5,7]. Among the main immune pathways, Th1 and Th17 responses are particularly important, with cytokines such as IL-17, IL-6, IL-1β, TNF-α, IFN-γ, and GM-CSF contributing to inflammatory amplification, blood–brain barrier disruption, and immune-cell migration into the central nervous system [8]. These processes promote demyelination, axonal injury, oxidative stress, and progressive neurodegeneration, supporting the concept of multiple sclerosis as both an inflammatory and neurodegenerative disease [8,9].

Periodontal disease is a chronic inflammatory condition initiated by the accumulation of oral biofilm and the development of oral dysbiosis, in which the balance between commensal and pathogenic microorganisms is disrupted. Although bacterial challenge is essential for disease initiation, periodontal tissue destruction is mainly driven by the host inflammatory response [1,5,10,11]. In susceptible individuals, dysbiotic biofilms stimulate the production of pro-inflammatory mediators such as IL-1β, IL-6, TNF-α, and IL-17, together with matrix metalloproteinases, oxidative stress pathways, and an imbalance in the RANKL/OPG system. These mechanisms promote connective tissue degradation, osteoclast activation, and progressive alveolar bone loss [12]. Importantly, periodontitis should not be regarded only as a localized oral disease, because inflammatory cytokines, bacterial products, and periodontal pathogens may disseminate into the systemic circulation, contributing to a chronic systemic inflammatory burden that may be relevant for immune-mediated diseases such as multiple sclerosis [13,14].

Several shared mechanisms may biologically connect periodontal disease and multiple sclerosis through a common pattern of chronic immune activation [1,11,15,16,17]. The Th17/IL-17 axis represents one of the most relevant convergent pathways [18,19,20,21] as IL-17 contributes to neutrophil recruitment, inflammatory amplification, osteoclast activation, and periodontal bone loss, while in multiple sclerosis it promotes autoimmune neuroinflammation, blood–brain barrier disruption, and immune-cell migration into the central nervous system [19,21,22,23,24]. Other pro-inflammatory mediators, including IL-1β, IL-6, and TNF-α, are also involved in both periodontal tissue destruction and CNS inflammatory activity [5,19,22,25,26]. In addition, oxidative stress and matrix metalloproteinases may contribute to tissue injury in both diseases, promoting extracellular matrix degradation in periodontitis and blood–brain barrier dysfunction or neural damage in MS [27,28]. Oral dysbiosis and periodontal pathogens may further sustain systemic immune activation through bacterial products and circulating inflammatory mediators, suggesting that the possible relationship between the two conditions is mediated by overlapping inflammatory pathways rather than by a direct causal mechanism [5,11,29].

Previous studies have mainly examined the relationship between multiple sclerosis and periodontal disease from isolated perspectives, including the prevalence of periodontitis in MS patients [1], oral manifestations associated with MS, oral microbiome alterations, and the possible association between specific periodontal pathogens and MS severity [5,11,30,31,32]. However, these approaches have not fully integrated the different levels of inflammation that may connect the two conditions. In particular, the combined evaluation of local periodontal inflammation, salivary biomarkers, serum or systemic inflammatory markers, and neurological inflammatory outcomes remains limited [11,33,34]. Moreover, many studies are affected by methodological heterogeneity, variable periodontal diagnostic criteria, small sample sizes, and incomplete adjustment for confounding factors such as smoking, disability, oral hygiene, medication, vitamin D status, and socioeconomic background [2,35]. As a result, the current literature supports biological plausibility and association but does not clearly establish causality. A systematic review focused on shared inflammatory pathways is therefore needed to critically synthesize the available evidence and clarify the extent to which periodontal inflammation may represent a relevant immunoinflammatory comorbidity in MS.

This systematic review investigates whether multiple sclerosis and periodontal disease share common immunoinflammatory pathways and how this potential connection is reflected through periodontal, salivary, systemic, microbial, and neurological markers. The aim is to synthesize the available evidence on the biological relationship between these two conditions and to clarify whether current findings support only an association, a plausible mechanistic link, or a possible causal interaction.

## 2. Materials and Methods

### 2.1. Study Design and Reporting Guidelines

This study was designed as a mixed-evidence systematic review investigating the shared immunoinflammatory pathways between periodontal disease and multiple sclerosis. The review was conducted and reported according to the PRISMA 2020 guidelines [36], using a predefined research question, a systematic literature search, explicit eligibility criteria, independent study selection, standardized data extraction, risk of bias assessment, and qualitative synthesis of the available evidence.

### 2.2. Search Strategy

A systematic literature search was performed in PubMed/MEDLINE, Cochrane Library, and Scopus. The search was limited to articles published between June 2020 and June 2026, in order to include recent evidence regarding the relationship between periodontal disease, multiple sclerosis, and shared inflammatory mechanisms. This interval was selected to reduce temporal heterogeneity while capturing recent developments in the proposed periodontal–multiple sclerosis inflammatory relationship. The search strategy combined terms related to multiple sclerosis, periodontal disease, oral dysbiosis, inflammatory mechanisms, systemic inflammation, oral microbiome, and neuroinflammation. Boolean operators were used, and the search strategy was adapted according to the syntax and indexing system of each database [37]. The main keyword groups used in the search are presented in Table 1.

The following search string was used as a model and adapted for each database: (“multiple sclerosis” OR “MS” OR “demyelinating disease” OR “neuroinflammatory disease”) AND (“periodontal disease” OR “periodontitis” OR “chronic periodontitis” OR “periodontal inflammation” OR “oral dysbiosis” OR “oral microbiome” OR “periodontal pathogens”) AND (“inflammation” OR “immune response” OR “immune dysregulation” OR “systemic inflammation” OR “oxidative stress” OR “matrix degradation” OR “bone resorption” OR “blood-brain barrier” OR “neuroinflammation”).

To ensure full transparency and reproducibility, the complete database-specific search strategies, including all search strings, indexing terms, filters, search dates, and field restrictions, have been deposited in the online repository associated with this review protocol (OSF: osf.io/qmex2) and are publicly accessible.

The database search was supplemented by manual screening of the reference lists of included articles and relevant reviews to identify additional eligible studies. The database search was performed independently by three operators: A.V.C., A.M.F., and I.R.F. Each operator was assigned to a different database. The retrieved records were subsequently compared, and any disagreements regarding search results, eligibility, or article relevance were resolved by majority decision.

### 2.3. Grey Literature Search

The grey literature was searched to reduce publication bias and identify potentially relevant unpublished or non-indexed evidence. Google Scholar, clinical trial registries, preprint servers, and institutional repositories were searched using simplified combinations of the main keywords. Records were limited to English-language documents published between June 2020 and June 2026 and were screened using the same eligibility criteria applied to database studies. Eligible records were appraised using the methodological tool corresponding to their study design and were included only if full text and sufficient methodological information were available.

### 2.4. Inclusion and Exclusion Criteria

Studies were selected according to predefined inclusion and exclusion criteria. Eligible articles included original human studies published in English between June 2020 and June 2026 that investigated multiple sclerosis, periodontal disease, oral dysbiosis, oral microbiome alterations, periodontal pathogens, inflammatory mechanisms, or systemic immune responses relevant to both conditions. Studies were considered for inclusion if they reported at least one of the following: periodontal parameters, oral or salivary biomarkers, serum/systemic inflammatory markers, oral microbiome findings, periodontal pathogen profiles, or MS-related clinical outcomes such as disease severity, disability, disease duration, relapse activity, or treatment status.

Studies were excluded if they were published outside the selected time interval, were not written in English, were not related to either multiple sclerosis or periodontal disease, or did not address inflammatory, microbial, periodontal, or neurological outcomes relevant to the research question. Case reports, editorials, letters to the editor, conference abstracts without full text, animal-only studies, in vitro-only studies, and articles without accessible full text were also excluded.

### 2.5. Study Selection Process

Following database searches and duplicate removal, titles and abstracts were independently screened by the three reviewers (V.C.A., A.M.F., and I.R.F.). Full-text eligibility assessment and data extraction were also performed independently by each one of the researchers. Any disagreements were resolved through discussion and, when necessary, by majority decision.

The study selection process was performed according to the PRISMA 2020 flow structure and included four main stages: identification, screening, eligibility assessment, and inclusion [36]. During the identification stage, records were retrieved from PubMed/MEDLINE, Cochrane Library, and Scopus, and additional records were identified through the manual screening of reference lists from relevant articles and reviews. After the removal of duplicate records, titles and abstracts were screened according to the predefined inclusion and exclusion criteria. Before title and abstract screening, records were identified through automated reference-management procedures as duplicate entries or records that were clearly outside the scope of the review based on bibliographic metadata, indexing terms, publication type, and predefined eligibility criteria. All flagged records were subsequently verified by the reviewers prior to exclusion to ensure consistency with the study selection criteria and to minimize the risk of selection bias.

During screening and eligibility assessment, clearly irrelevant records were excluded, and the remaining full-text articles were assessed for final inclusion. Studies were excluded if they did not meet the inclusion criteria, were not written in English, were published outside the selected period, lacked accessible full text, or did not address multiple sclerosis and periodontal disease in relation to inflammatory, microbial, periodontal, or neurological outcomes. The final included studies were selected for qualitative synthesis, and the complete process was summarized using a PRISMA flow diagram, including records identified, duplicates removed, records screened, reports assessed for eligibility, excluded studies with reasons, and studies included in the final synthesis [36]. The selection process was independently performed by V.C.A., A.M.F., and I.R.F., and disagreements were resolved by majority decision.

### 2.6. Quality and Risk of Bias Assessment

The methodological quality and risk of bias of the included studies were assessed according to their study design. Observational studies, including cohort and case–control studies, were evaluated using the Newcastle–Ottawa Scale [38], while cross-sectional studies were assessed using the Joanna Briggs Institute critical appraisal checklist [39]. The systematic reviews and meta-analyses quality was assessed using AMSTAR 2 [40]. The assessment focused on participant selection, comparability of study groups, exposure and outcome measurement, control of confounding factors, completeness of data reporting, clarity of statistical analysis, and overall methodological rigor. To improve the methodological robustness of the final synthesis, studies scoring below 6 points on the NOS or JBI assessment tools were excluded from the qualitative synthesis. Similarly, systematic reviews and meta-analyses classified as critically low confidence according to AMSTAR 2 were excluded. These predefined thresholds were applied to minimize the influence of studies with substantial methodological limitations and to ensure that the final synthesis was based on evidence of at least moderate methodological quality.

Each study was independently evaluated by V.C.A., A.M.F., and I.R.F., and any disagreements were resolved by majority decision. The results of the quality assessment were used to interpret the strength of the available evidence and to distinguish between methodological limitations, biological plausibility, association, and possible causal inference.

### 2.7. Data Extraction

Data extraction was performed using a standardized form developed for this systematic review. For each included study, the following information was collected: author, year of publication, study type, disease or condition investigated, and main findings.

The disease or condition investigated referred to the main focus of each study, such as multiple sclerosis, periodontal disease, oral dysbiosis, oral microbiome alterations, periodontal pathogens, neuroinflammation, or the relationship between periodontal and neurological inflammatory mechanisms. This category was used to clarify whether the study directly evaluated both conditions or addressed only one disease in relation to inflammatory pathways relevant to the review question.

The main findings included the principal results reported by each study, especially those related to shared inflammatory mechanisms, systemic inflammation, oral microbial changes, periodontal involvement in MS patients, or associations between periodontal status and MS-related outcomes. The extracted data were organized in evidence tables to facilitate comparison between studies and to support the qualitative synthesis. Data extraction was performed independently by V.C.A., A.M.F., and I.R.F., and discrepancies were resolved by majority decision.

Because both primary studies and systematic reviews were eligible for inclusion, the two types of evidence were assigned distinct roles in the qualitative synthesis. Primary studies were considered the principal source of direct clinical, periodontal, microbial, and biomarker findings, whereas systematic reviews and meta-analyses were used mainly to contextualize the broader mechanistic pathways and to identify areas of consistency or uncertainty across the literature. Primary and secondary publications were not treated as equivalent or independent quantitative units, and no effect estimates from the two evidence levels were statistically pooled. When an included systematic review contained primary studies that were also included directly or were represented in another review, the resulting overlap was considered during interpretation. Repeated reporting of the same underlying primary evidence was treated as corroborative rather than as additional independent evidence and did not, by itself, increase the certainty assigned to an inflammatory pathway.

### 2.8. Strength of Evidence Assesment

The strength of evidence was assessed using a modified GRADE approach, considering that the synthesis of findings was organized by inflammatory pathway rather than by individual article. Because this review focused primarily on mechanistic, observational, and secondary evidence rather than intervention-specific outcomes, GRADE was applied narratively to each identified immunoinflammatory pathway. The assessment was based on five predefined domains: the risk of bias and methodological quality of the contributing studies, consistency of findings across the available literature, directness of the evidence linking periodontal disease and multiple sclerosis, precision and robustness of the reported findings, and potential influence of confounding factors or other sources of bias.

Given the heterogeneous nature of the included evidence, formal quantitative GRADE upgrading and downgrading procedures were not applied. Instead, domain-level judgments were integrated qualitatively to determine the overall certainty of evidence for each pathway. Pathways supported predominantly by observational, mechanistic, or indirect evidence, as well as those exhibiting important methodological limitations, inconsistency of findings, imprecision, or substantial risk of bias, were downgraded accordingly. Based on these criteria, the certainty of evidence for each pathway was classified as high, moderate, low, or very low [41].

### 2.9. Protocol Registration

The protocol of this systematic review was registered on the Open Science Framework (OSF) under the registration identifier osf.io/qmex2 in order to ensure methodological transparency and reproducibility and reduce the risk of selective reporting.

### 2.10. Image Generation

Generative artificial intelligence (GenAI) tools were used to assist in the conceptual development and visual design of schematic figures included in this manuscript. ChatGPT (version 5.5, OpenAI, San Francisco, CA, USA) was used to support figure planning, organization of visual elements, and graphical layout concepts. All figures were subsequently reviewed, modified where necessary, and scientifically validated by the authors.

## 3. Results

### 3.1. Database Search Results

The systematic database search was conducted in PubMed/MEDLINE, Cochrane Library, and Scopus. A total of 1233 records were identified: 101 records from PubMed/MEDLINE, 18 from Cochrane Library, and 1114 from Scopus. Before screening, 87 duplicate records were removed, 322 records were identified through automated reference-management procedures as duplicate entries or records that were clearly outside the scope of the review and were subsequently verified by the reviewers prior to exclusion, and 578 records were excluded because they referred to other diseases or were outside the scope of the review.

After this stage, 246 records were screened based on title and abstract. Of these, 137 records were excluded because they were not relevant to the research question. A total of 109 reports were sought for retrieval, of which 17 reports could not be retrieved due to the unavailability of full text. Therefore, 92 full-text reports were assessed for eligibility.

**Figure 1:**
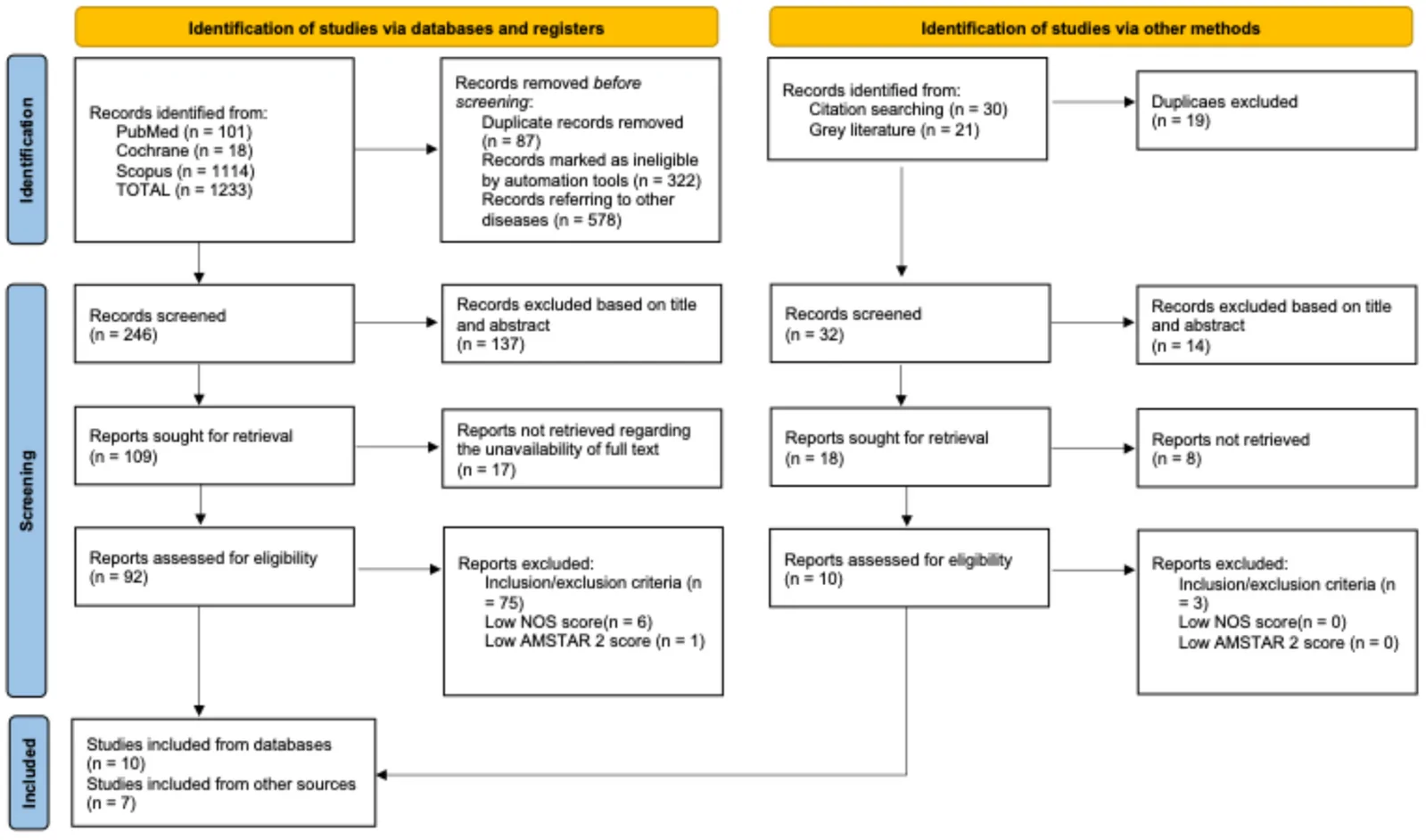
PRISMA flow chart of the study selection process, including records identified through databases and other sources. The flow diagram was generated using the PRISMA 2020 interactive template available at https://www.prisma-statement.org/prisma-2020-flow-diagram (accessed on 30 June 2026) [42].

Following full-text assessment, 75 reports were excluded because they did not meet the inclusion and exclusion criteria, six studies did not meet the minimum Newcastle–Ottawa Scale quality threshold, while one systematic review was classified as critically low confidence according to AMSTAR 2. Finally, 10 studies identified through database searching were included in the qualitative synthesis. The complete study selection process is presented in Figure 1.

### 3.2. Results from Other Sources

In addition to the database search, 30 records were identified through other methods, specifically by citation searching of relevant articles and reviews. Before screening, 19 duplicate records were excluded, leaving 32 records for title and abstract screening. During screening, 14 records were excluded because they were not relevant to the research question. A total of 18 reports were then sought for retrieval, of which 8 reports could not be retrieved. Therefore, 10 full-text reports were assessed for eligibility.

After full-text assessment, 3 reports were excluded because they did not meet the predefined inclusion and exclusion criteria. No studies from this group were excluded due to a low Newcastle–Ottawa Scale score or a low AMSTAR 2 score. Finally, 7 studies identified through other sources were included in the qualitative synthesis. The complete selection process for studies identified through other sources is presented together with the database search results in Figure 1.

### 3.3. Grey Literature Search Results

The grey literature search identified 21 records, including 7 doctoral theses, 5 conference abstracts, 2 preprints, 2 institutional reports, and 5 trial registry records. All records were screened according to the same eligibility criteria applied to the database search, including language, publication period, topic relevance, full-text accessibility, and methodological suitability. After screening and eligibility assessment, none of the 21 grey literature records were included in the qualitative synthesis. The excluded records were not considered eligible because they did not provide sufficient methodological information, did not directly address the relationship between multiple sclerosis and periodontal disease through inflammatory or microbial mechanisms, were not sufficiently aligned with the predefined research question, or did not meet the inclusion criteria. The complete follow-up of these records is presented in the PRISMA flow chart.

### 3.4. Risk of Bias Assessment

The methodological quality and risk of bias of the included studies were assessed using tools appropriate to each study design. The Newcastle–Ottawa Scale was applied to observational cohort and case–control studies [38], AMSTAR 2 was used for systematic reviews and meta-analyses [40], and the Joanna Briggs Institute Critical Appraisal Checklist was applied to cross-sectional studies [39].

The Newcastle–Ottawa Scale was used to assess the methodological quality of observational cohort and case–control studies. This tool evaluates three major domains: selection of study participants, comparability of study groups, and assessment of outcome or exposure, with a maximum total score of 9 points. Studies scoring 7–9 points were classified as having a low risk of bias, those scoring 5–6 points as having a moderate risk of bias, and those scoring below 5 points as having a high risk of bias [38]. In the present review, the Newcastle–Ottawa Scale was applied to one prospective observational cohort study. Abdullah (2025) [19] obtained a score of 6 out of 9 points, corresponding to a moderate risk of bias. The main limitations identified were the absence of community-derived controls for the non-exposed group and incomplete follow-up reporting, which may have introduced selection and attrition bias. The Newcastle–Ottawa Scale assessment is summarized in Table 2.

AMSTAR 2 was used to evaluate the methodological quality of the systematic reviews and meta-analyses included in this review. This tool includes 16 items, of which seven are considered critical domains: protocol registration before the review, comprehensive literature search, justification for excluded studies, formal risk of bias assessment, appropriateness of meta-analytical methods, consideration of risk of bias when interpreting results, and assessment of publication bias. Overall confidence was classified as high, moderate, low, or critically low according to the presence and severity of methodological weaknesses [40]. The AMSTAR 2 assessment is presented in Table 3.

AMSTAR 2 was applied to thirteen systematic reviews and meta-analyses. One study, De Picker et al. (2023) [43], achieved a high-confidence rating. Three studies were classified as having moderate methodological confidence: Gavrilă et al. (2026) [21], Arcas et al. (2024, salivary biomarkers) [11], and Arcas et al. (2024, miR-150) [5]. These studies showed no major critical weaknesses, although some non-critical limitations were identified, mainly related to incomplete reporting of funding sources of included primary studies, absence of a registered protocol, or limited discussion of methodological heterogeneity. The remaining nine systematic reviews were rated as having low methodological confidence. Zapata-Acevedo et al. (2022) [20], Bagnato et al. (2025) [44], Dhungana et al. (2025) [24], and Arriaga et al. (2026) [45] were rated low mainly because publication bias was not formally assessed. Smusz et al. (2025) [7] and Li et al. (2026) [46] were rated low because the influence of risk of bias, heterogeneity, or methodological limitations on the interpretation of results was insufficiently addressed. García-Rios et al. (2025) [32], Martínez-Martínez et al. (2025) [47], and Salhi et al. (2023) [23] were rated low due to the absence of a formal risk of bias assessment using a validated tool. Santhanakrishnan et al. (2026) [22] was rated low because excluded studies were not listed with reasons and publication bias was not formally evaluated. These limitations were considered when interpreting the strength of evidence regarding the shared inflammatory pathways between periodontal disease and multiple sclerosis.

**Table 3:** AMSTAR 2 critical domains, methodological quality assessment of systematic reviews and meta-analyses included in the review.

Author, Year	Item 2	Item 4	Item 7	Item 9	Item 11	Item 13	Item 15	Overall Confidence
De Picker et al., 2023 [43]	Y	PY	Y	PY	Y	PY	Y	High
Arriaga et al., 2026 [45]	N	N	Y	N	N/A	N/A	N/A	Low
Zapata-Acevedo et al., 2022 [20]	Y	N	N	Y	N/A	N/A	N/A	Low
García-Rios et al., 2025 [32]	Y	N	PY	Y	N/A	N/A	N/A	Low
Bagnato et al., 2025 [44]	N	PY	N	N	N/A	N/A	N/A	Low
Gavrilă et al., 2026 [21]	Y	N	N	Y	N/A	N/A	N/A	Moderate
Santhanakrishnan et al., 2026 [22]	N	Y	N	Y	N/A	N/A	N/A	Low
Arcas et al., 2024 (saliva) [11]	Y	Y	N	Y	Y	PY	Y	Moderate
Arcas et al., 2024 (miR-150) [5]	Y	Y	N	Y	Y	Y	Y	Moderate
Martínez-Martínez et al., 2025 [47]	Y	N	N	Y	N/A	PY	N	Low
Salhi et al., 2023 [23]	Y	N	PY	Y	N/A	PY	N	Low
Dhungana et al., 2025 [24]	Y	N	N	Y	N/A	PY	PY	Low
Smusz et al., 2025 [7]	Y	N	PY	N	N	N	Y	Low
Li et al., 2026 [46]	Y	N	N	Y	Y	PY	Y	Low

Item 1—PICO components; Item 2—protocol registration; Item 3—study design justification; Item 4—comprehensive literature search; Item 5—duplicate study selection; Item 6—duplicate data extraction; Item 7—excluded studies list; Item 8—description of included studies; Item 9—risk of bias assessment; Item 10—funding sources of included studies; Item 11—appropriate meta-analysis methods; Item 12—impact of risk of bias on meta-analysis; Item 13—risk of bias considered in interpretation; Item 14—explanation of heterogeneity; Item 15—publication bias assessment; and Item 16—conflict of interest. Y means yes, N means no, N/A means not addressed, PY means partial yes.

The Joanna Briggs Institute Critical Appraisal Checklist for Cross-Sectional Studies was applied to evaluate the methodological quality of cross-sectional observational studies. This checklist assesses whether inclusion criteria were clearly defined, whether study subjects and settings were adequately described, whether exposure and outcome measurements were valid and reliable, whether confounding factors were identified and addressed, and whether appropriate statistical analysis was used. Each item was scored as yes, no, unclear, or not applicable, with a maximum score of 8 points. Studies scoring 7–8 points were classified as having a low risk of bias, those scoring 5–6 points as having a moderate risk of bias, and those scoring 4 or below as having a high risk of bias [39]. The JBI assessment is summarized in Table 4.

The JBI checklist was applied to two cross-sectional studies. Zilberstein et al. (2025) [48] achieved a score of 8 out of 8 points, corresponding to a low risk of bias, reflecting rigorous patient selection criteria, validated measurement tools, calibrated examiners, and appropriate multivariate statistical analysis. Nie and Lin (2026) [49] also achieved a score of 8 out of 8 points and was classified as having a low risk of bias, supported by the use of next-generation 16S rRNA sequencing, WHO-standardized oral examination criteria with inter-examiner calibration, and multivariate logistic regression with adjustment for relevant confounders.

Overall, the risk of bias assessment showed that the observational evidence was limited but generally acceptable, while most systematic reviews and meta-analyses presented low overall confidence according to AMSTAR 2. The most important methodological limitations were related to incomplete assessment of publication bias, lack of protocol registration, insufficient reporting of excluded studies, and limited consideration of risk of bias when interpreting results. These limitations were taken into account when interpreting the strength of evidence regarding the shared inflammatory pathways between periodontal disease and multiple sclerosis.

**Figure 2:**
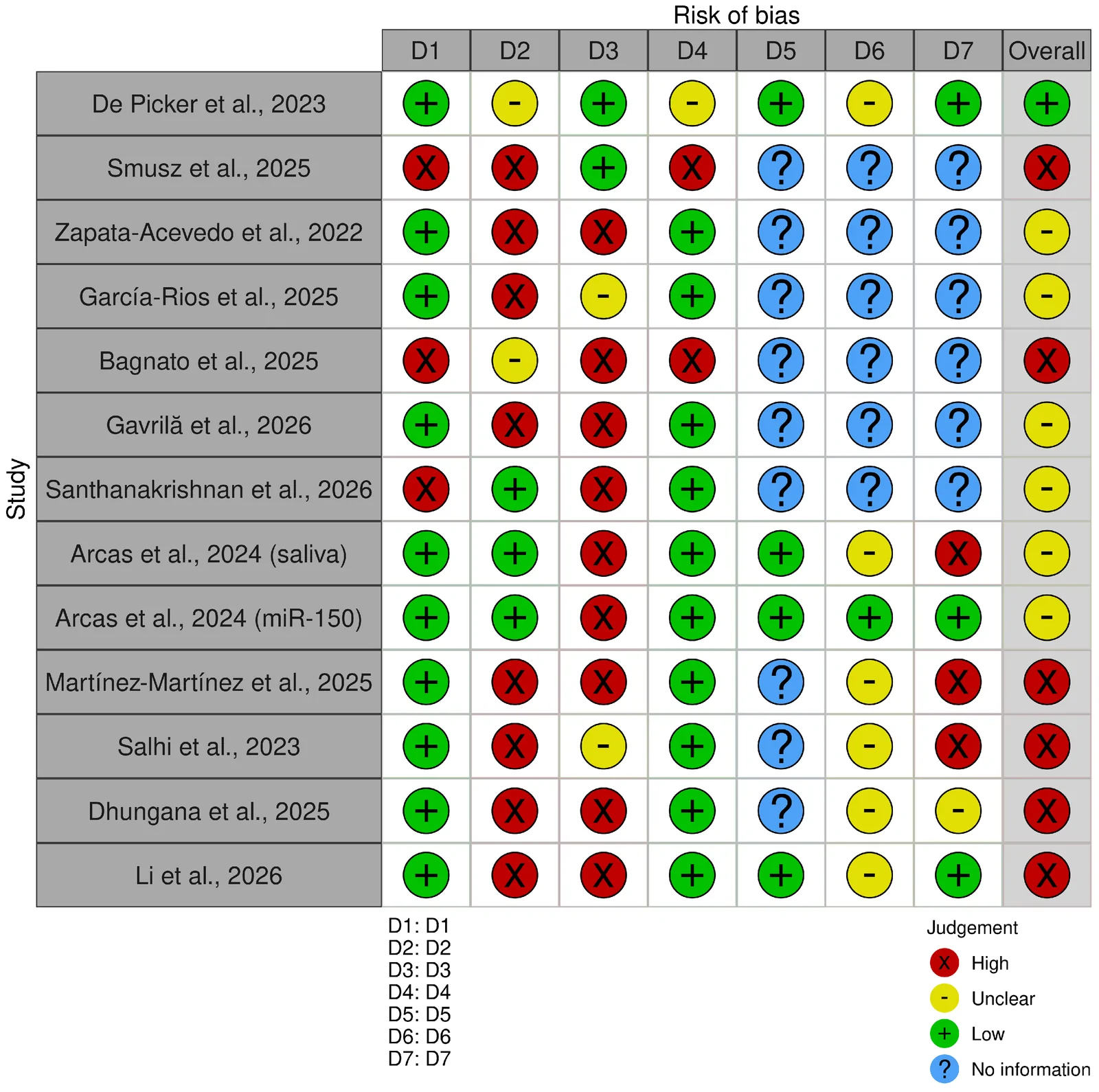
Traffic light plot of methodological quality assessment for included systematic reviews and meta-analyses [5,7,11,20,21,22,23,24,32,43,44,46,47].

**Figure 3:**
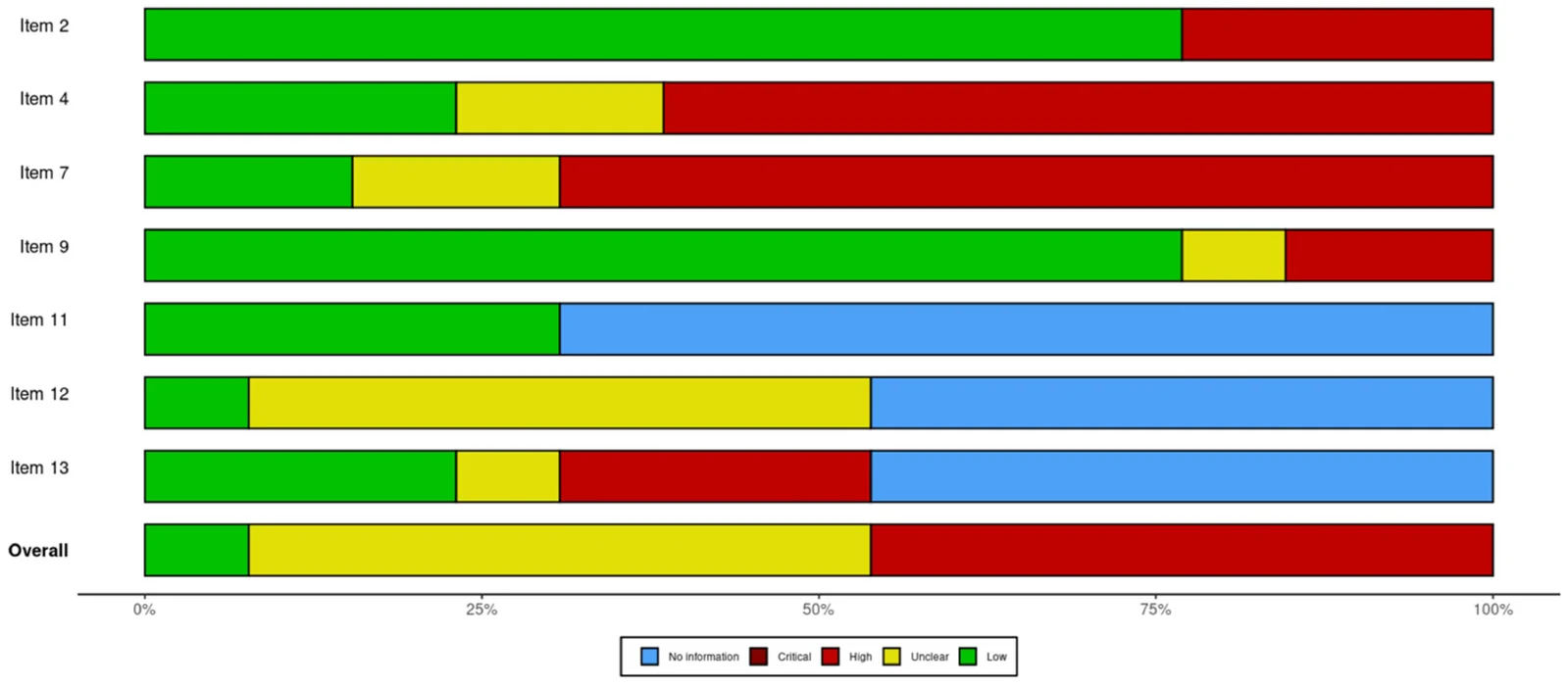
Summary plot of methodological quality assessment for included systematic reviews and meta-analyses.

**Figure 4:**
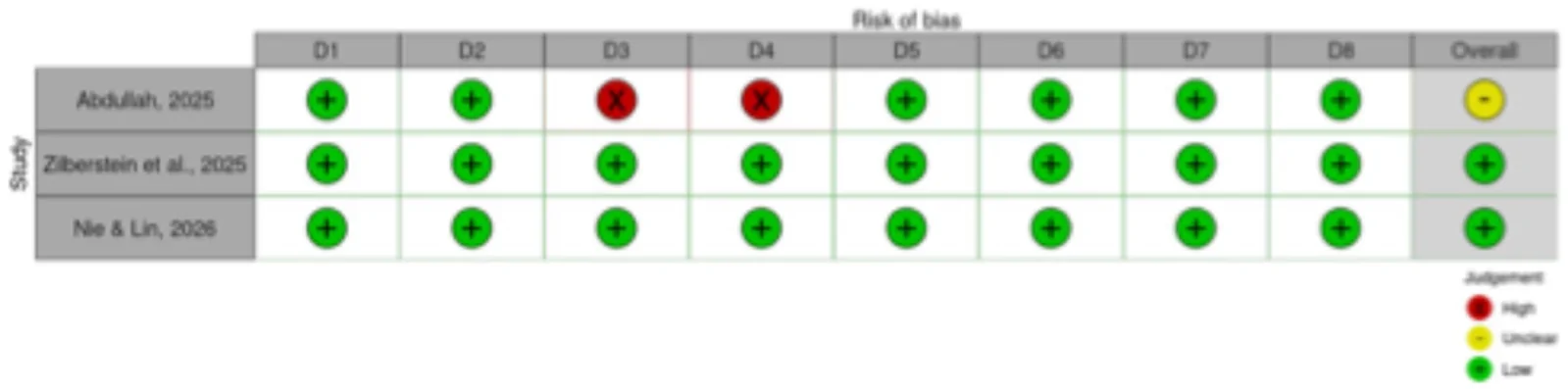
Traffic light plot of risk of bias assessment for observational studies using NOS and JBI tools [9,48,49].

**Figure 5:**
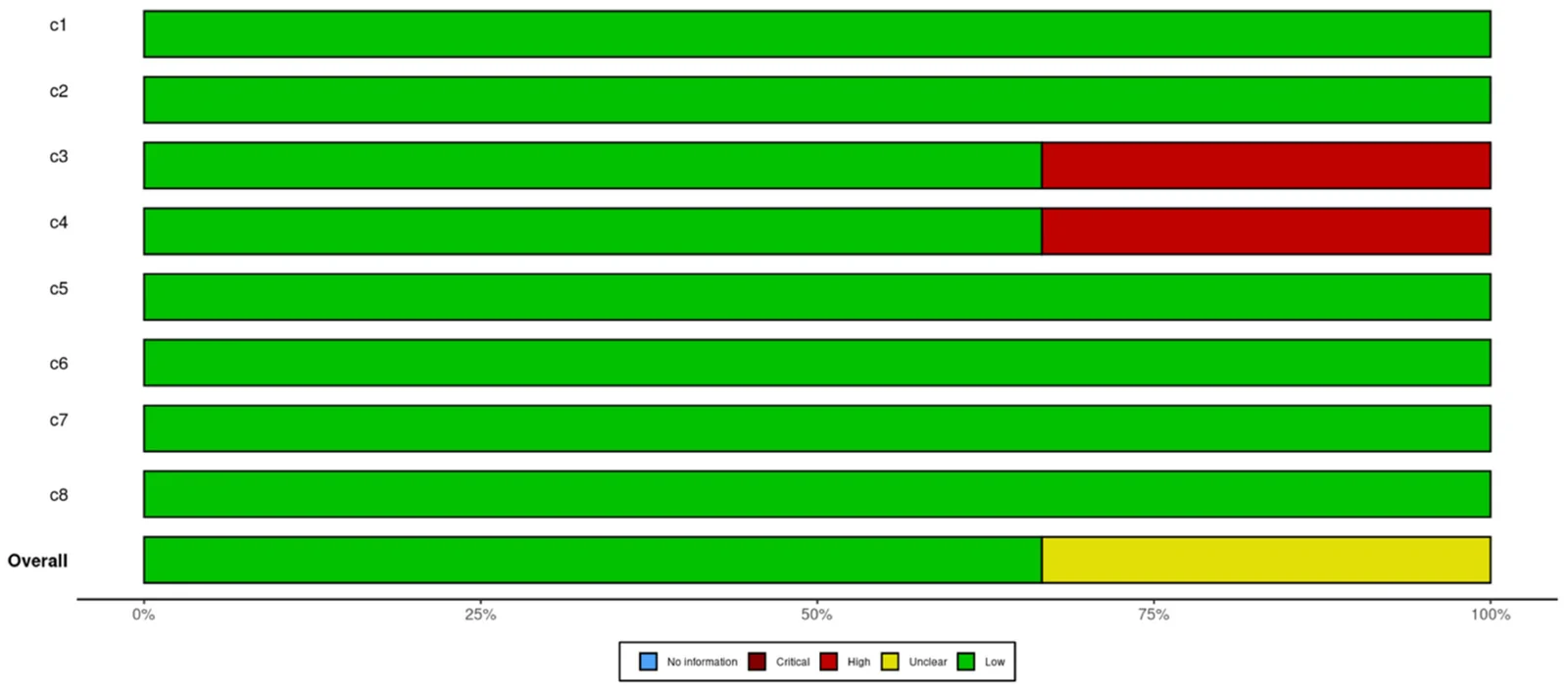
Summary plot of risk of bias assessment for observational studies using NOS and JBI tools.

To facilitate interpretation, the results were graphically visualized using the robvis online visualization tool [50]. For the AMSTAR 2 assessment, the traffic light plot and summary plot are presented in Figure 2 and Figure 3, respectively. For the observational studies assessed using NOS and JBI, the corresponding traffic light plot and summary plot are presented in Figure 4 and Figure 5, respectively.

### 3.5. Excluded Articles

Articles excluded during the screening and eligibility assessment stages were documented in order to ensure transparency and reproducibility of the selection process. Exclusions were performed according to the predefined inclusion and exclusion criteria described in the Section 2. The main reasons for exclusion included lack of relevance to the research question, absence of data regarding multiple sclerosis or periodontal disease, absence of inflammatory, microbial, periodontal, or neurological outcomes, publication outside the selected time interval, unavailability of full text, insufficient methodological information, and low methodological quality according to the corresponding appraisal tool.

A complete list of all excluded articles, including the stage at which each article was excluded and the specific reason for exclusion, is available in the online repository linked to the OSF registration of this review under the registration code osf.io/qmex2.

### 3.6. Synthesis of Findings

**Figure 6:**
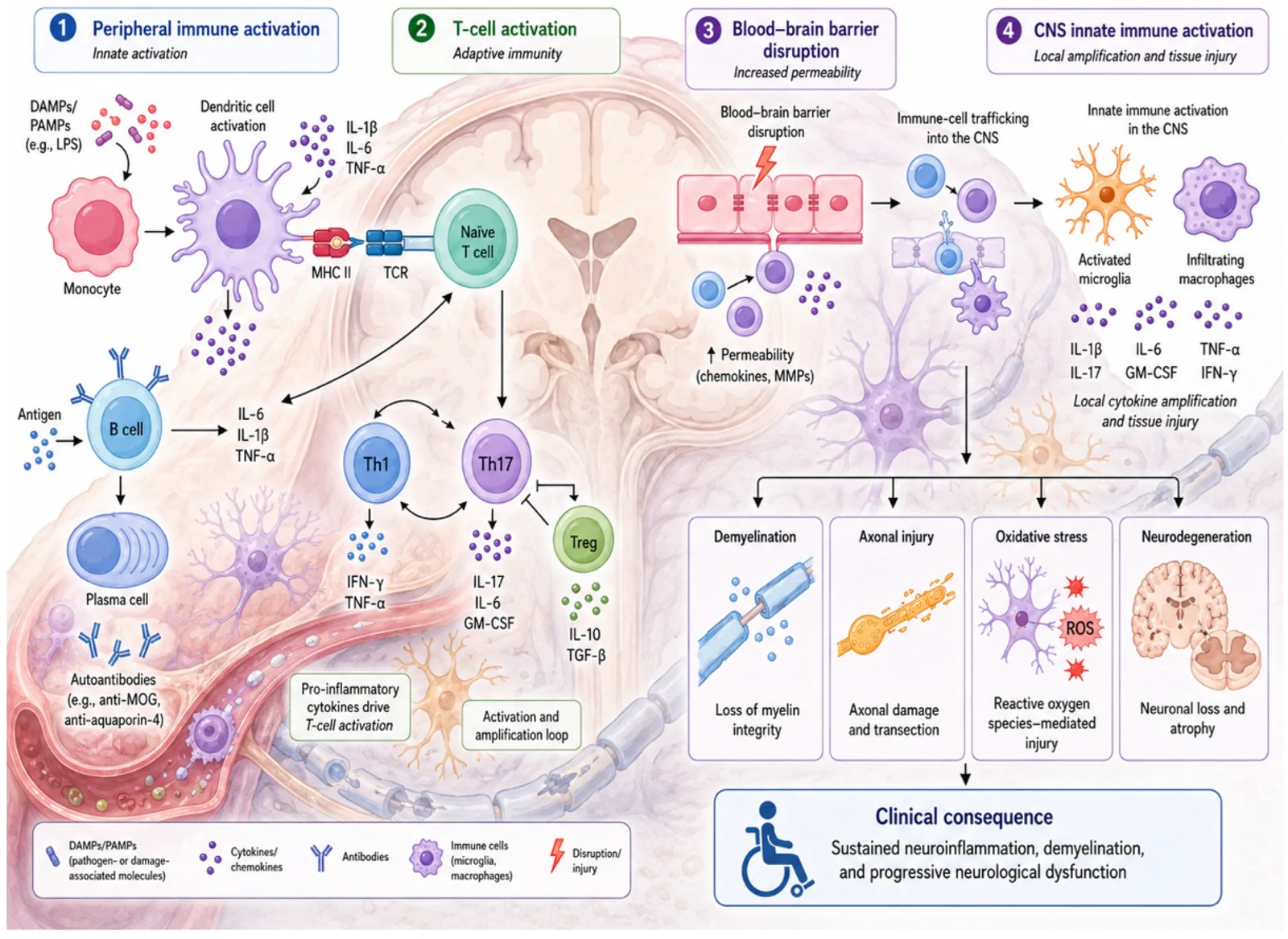
Inflammatory pathways involved in multiple sclerosis pathogenesis (Image generated using ChatGPT (OpenAI, GPT-4, 2024/2025)) [51].

**Figure 7:**
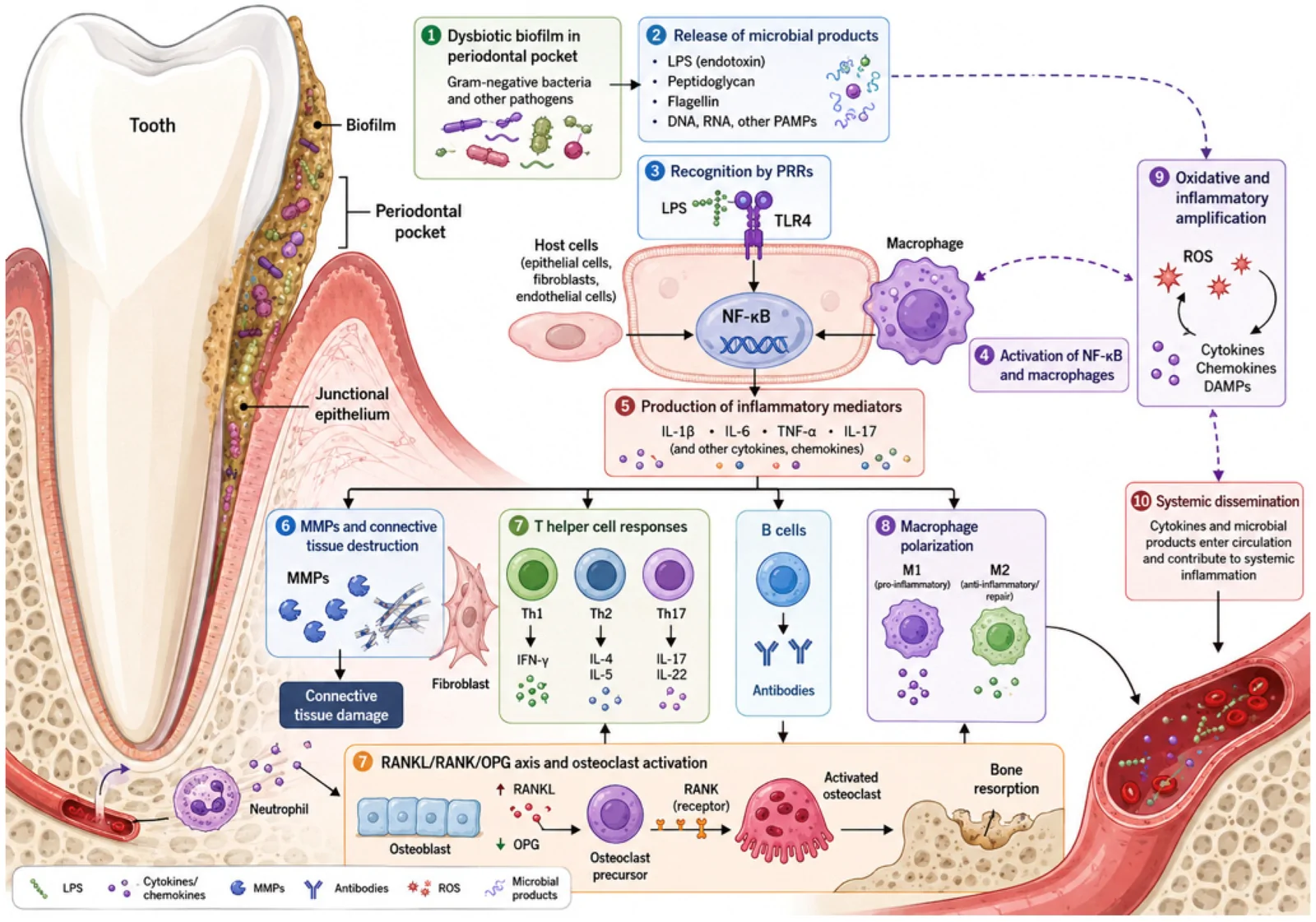
Inflammatory pathways involved in periodontal disease pathogenesis (Image generated using ChatGPT (OpenAI, GPT-4, 2024/2025)) [51].

**Figure 8:**
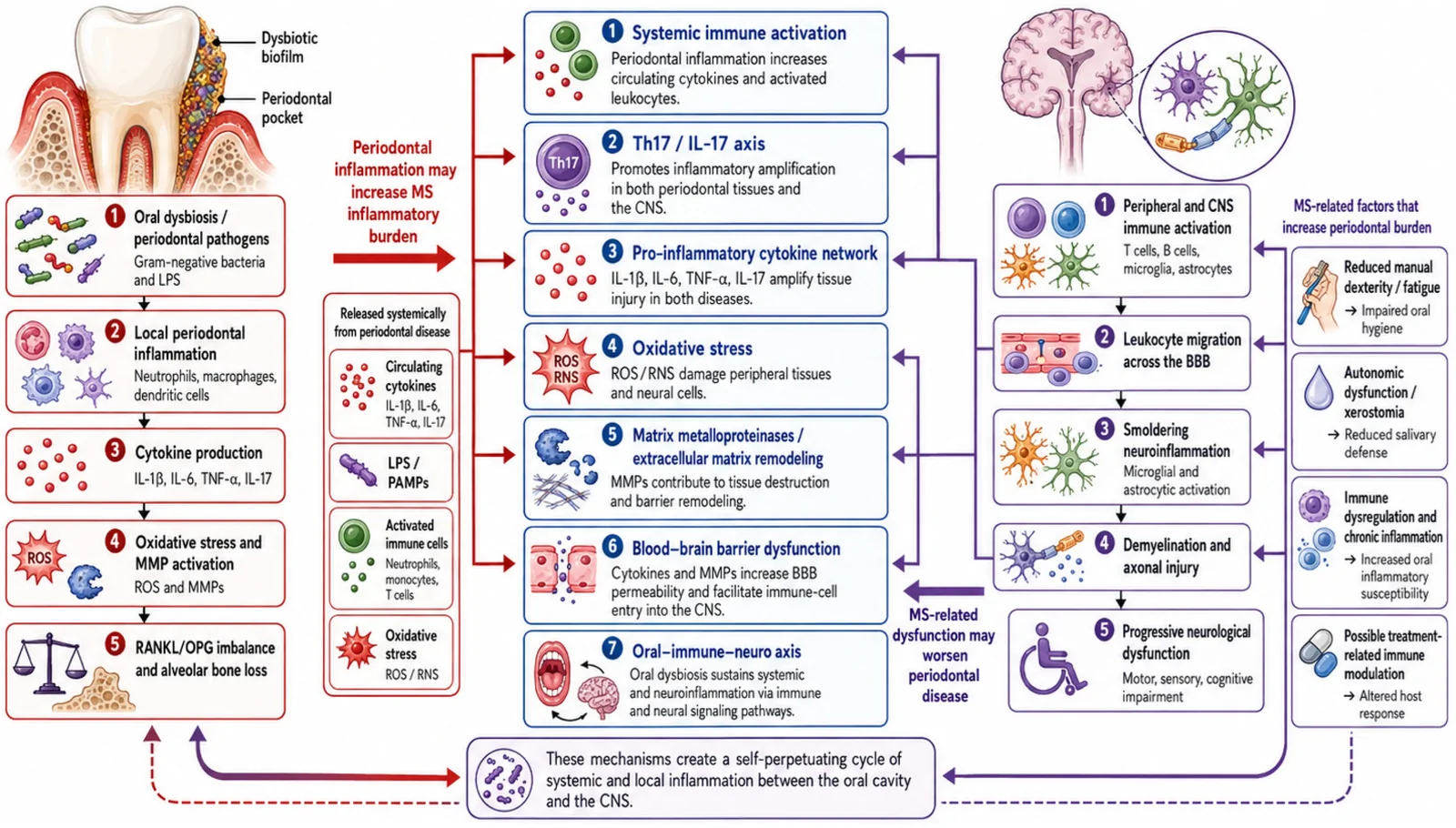
Bidirectional immunoinflammatory interaction between periodontal disease and multiple sclerosis(Image generated using ChatGPT (OpenAI, GPT-4, 2024/2025)) [51].

The synthesis of findings was organized according to the main immunoinflammatory pathways identified across the included evidence. Rather than presenting the results study by study, the findings were grouped into mechanistic categories in order to clarify how periodontal disease and multiple sclerosis may interact through convergent biological processes. The MS-related inflammatory pathways are visually summarized in Figure 6, the periodontal inflammatory pathways are presented in Figure 7, while the common pathways and their interactions are presented in Figure 8. The characteristics and main findings of the included studies are reported in Table 5. The shared immunoinflammatory mechanisms are summarized in Table 6, the distribution of inflammatory evidence across biological compartments is presented in Table 7, and the proposed bidirectional interactions between periodontal disease and multiple sclerosis are shown in Table 8.

The most consistently reported shared mechanism was cytokine-mediated systemic inflammation [5,7,11,19,20,21,22,23,24,32,45,46,47,48]. Several studies identified TNF-α, IL-1β, IL-6, and IL-17 as central mediators linking periodontal inflammation with neuroinflammatory activity [22,23]. In periodontal disease, these cytokines are generated locally in response to dysbiotic biofilm and periodontal pathogens, contributing to immune-cell recruitment, connective tissue degradation, and alveolar bone resorption [23,24,47,49]. In multiple sclerosis, the same cytokines participate in peripheral immune activation, endothelial dysfunction, blood–brain barrier permeability, and CNS inflammatory amplification [21,43,44,47]. Abdullah [19] reported that patients with concurrent multiple sclerosis and periodontitis showed elevated systemic IL-6 and TNF-α levels, supporting the relevance of systemic cytokine burden as a shared inflammatory mechanism. Santhanakrishnan et al. [22] further described how periodontal-derived IL-1β, IL-6, and TNF-α may impair blood–brain barrier tight junctions and facilitate immune-cell transmigration into the CNS. These findings suggest that periodontal inflammation may contribute to a systemic cytokine milieu capable of amplifying the neuroinflammatory environment of multiple sclerosis.

NF-κB signaling also emerged as a convergent molecular pathway. In periodontal disease, bacterial products such as lipopolysaccharide activate pattern-recognition receptors, particularly TLR2 and TLR4, leading to NF-κB activation and subsequent production of inflammatory cytokines, adhesion molecules, and matrix-degrading enzymes [22,23,24,46]. In multiple sclerosis, NF-κB signaling contributes to inflammatory cell activation, endothelial dysfunction, matrix metalloproteinase expression, and blood–brain barrier disruption [3,19,20,21,22,28,44]. Zapata-Acevedo et al. [20] emphasized the role of NF-κB-driven MMP-3 and MMP-9 upregulation in MS-related extracellular matrix degradation, while Dhungana et al. [24] and Li et al. [46] described similar NF-κB-mediated inflammatory responses induced by periodontal pathogens in systemic vascular disease. Although some of these findings are derived from non-MS inflammatory models, they support the concept that NF-κB represents a biologically plausible molecular bridge between periodontal infection, systemic inflammation, and CNS immune activation.

Blood–brain barrier disruption represented a key structural mechanism through which peripheral inflammation may influence CNS pathology [19,20,22,23,24,44,45,46,47]. In multiple sclerosis, increased blood–brain barrier permeability allows peripheral immune cells and inflammatory mediators to enter the CNS, promoting demyelination and neuroaxonal injury [9,20,22,44]. Zapata-Acevedo et al. [20] described how cytokine-driven matrix metalloproteinase activity degrades extracellular matrix components and tight junction proteins, including claudin-5, occludin, and ZO-1. Santhanakrishnan et al. [22] further suggested that periodontal pathogens and their endotoxins may downregulate tight junction proteins through cytokine-mediated signaling. Therefore, periodontal inflammation may contribute indirectly to neuroinflammation by increasing systemic cytokine and MMP activity, which may weaken endothelial and blood–brain barrier integrity.

The Th17/Treg axis was another major shared pathway [19,20,21,22,24,45,48]. In multiple sclerosis, Th17 polarization contributes to autoimmune neuroinflammation, blood–brain barrier disruption, and immune-cell migration into the CNS [5,19,21,22,48]. In periodontal disease, Th17 responses promote neutrophil recruitment, cytokine amplification, osteoclast activation, and alveolar bone loss [22,23,24,32]. Gavrilă et al. [21] reported increased IL-17 and Th17 activity in MS, particularly during inflammatory disease phases, while Zapata-Acevedo et al. [20] described mechanisms facilitating Th17 transmigration across the blood–brain barrier. Zilberstein et al. [48] connected concurrent MS and periodontitis with altered metabolic profiles associated with Th17/Treg imbalance, and García-Rios et al. [32] identified shared immune-regulatory signatures between the two conditions. These findings suggest that periodontal dysbiosis may contribute to systemic adaptive immune imbalance, potentially amplifying the Th17-driven inflammatory processes involved in MS.

Microglial activation and smoldering neuroinflammation were mainly MS-specific mechanisms, but they may be influenced by systemic periodontal inflammation [21,22,23,43,44]. De Picker et al. [43] showed that TSPO-PET imaging detects persistent microglial and astrocytic activation in MS, supporting the presence of chronic CNS-intrinsic innate immune activity. Bagnato et al. [44] further described smoldering lesions characterized by iron-laden microglia and macrophages, which contribute to progressive neuroaxonal injury independently of acute relapse activity. Periodontal inflammation may influence this pathway indirectly through circulating cytokines, bacterial products, and oxidative stress. Santhanakrishnan et al. [22] proposed that periodontal LPS and systemic inflammatory mediators may activate microglia through TLR4-dependent mechanisms, while Salhi et al. [23] described pathogen-associated microglial activation through pathways involving gingipains and pro-inflammatory glial polarization. These findings support the hypothesis that periodontal inflammation may act as a peripheral amplifier of CNS innate immune activation.

Oral dysbiosis and the oral–gut–brain axis represented a distinct pathway cluster linking local periodontal pathology with systemic and neurological inflammation [7,24,47,48,49]. Nie and Lin [49] described oral dysbiosis characterized by reduced microbial diversity and expansion of potentially pathogenic taxa, while Zilberstein et al. [48] emphasized the gum–gut axis and its possible role in systemic immune modulation. Smusz et al. [7] identified tryptophan metabolism and the kynurenine pathway as relevant metabolic-inflammatory mechanisms in MS, suggesting that microbial and metabolic dysregulation may influence neuroinflammation. In this context, oral pathobionts may translocate to the gut, alter mucosal immune responses, and contribute to systemic inflammatory signaling. These findings position oral microbiome dysbiosis as a potentially modifiable factor within the broader oral–immune–neuroinflammatory axis.

Oxidative and metabolic stress also appeared as shared mechanisms [7,21,22,24]. In periodontal disease, oxidative stress contributes to connective tissue injury, inflammatory amplification, and periodontal destruction [24,32,46,49]. In multiple sclerosis, oxidative and nitrosative stress contribute to mitochondrial dysfunction, demyelination, axonal injury, and neurodegeneration [22,23,48]. Smusz et al. [7] described several metabolic-inflammatory pathways in MS, including altered tryptophan catabolism, eicosanoid imbalance, reduced short-chain fatty acid signaling, and impaired nitric oxide regulation. Santhanakrishnan et al. [22] connected periodontal inflammation with oxidative injury through salivary malondialdehyde and total oxidative stress, supporting the idea that oxidative damage may be reflected across oral and systemic compartments. Thus, oxidative stress may represent a shared downstream pathway linking periodontal tissue destruction with neurodegenerative progression.

Matrix metalloproteinases and extracellular matrix remodeling formed another relevant mechanistic bridge [11,20,22,23,24,46]. In periodontal disease, MMPs degrade collagen and extracellular matrix components, contributing to periodontal attachment loss and connective tissue destruction [20,23,24]. In multiple sclerosis, MMPs are involved in blood–brain barrier disruption, basement membrane degradation, immune-cell trafficking, and tissue remodeling [20,22]. Zapata-Acevedo et al. [20] highlighted MMP-3 and MMP-9 as key mediators of extracellular matrix disruption in MS, while Arcas et al. [11] reported salivary MMPs as potential biomarkers linking oral inflammation with systemic and neurological inflammatory activity. This suggests that matrix remodeling may represent a common tissue-destructive process operating in both periodontal and CNS inflammatory compartments.

Bone-immune signaling, particularly through the RANKL/OPG axis, was mainly relevant to periodontal disease but also reflected systemic immune activation. In periodontitis, inflammatory cytokines increase RANKL expression and reduce the protective effects of OPG, promoting osteoclast activation and alveolar bone resorption [9,22,24,32,46,48,49]. García-Rios et al. [32] emphasized the involvement of IL-6 and TNF-α in RANKL/OPG dysregulation, suggesting that periodontal bone loss may reflect broader cytokine-driven immune activation. Although RANKL/OPG signaling is not a central structural pathway in MS, the cytokine environment that drives this axis overlaps with systemic inflammatory mechanisms involved in neuroinflammation [19].

Complement activation was identified as another bridging innate immune mechanism [21,23,24,32]. García-Rios et al. [32] reported complement factor I among shared molecular signatures between MS and periodontal disease, while Gavrilă et al. [21] described complement activation as part of the MS inflammatory biomarker profile. Salhi et al. [23] also discussed complement activation induced by periodontal pathogens, particularly through mechanisms involving *Porphyromonas gingivalis*. These findings suggest that complement dysregulation may connect periodontal innate immune responses with CNS glial activation and neuroinflammatory amplification.

Finally, several studies linked these inflammatory pathways to neuroaxonal injury and structural CNS damage [5,19,21,22,23,44,45,47]. Gavrilă et al. [21] described serum NfL and GFAP as biomarkers of neuroaxonal injury and reactive astrogliosis in MS, while Abdullah [19] reported higher sNfL levels in MS patients with concurrent active periodontitis. Bagnato et al. [44] associated smoldering neuroinflammation with chronic neuroaxonal loss, and Arriaga et al. [45] provided evidence from neurodegenerative disease models suggesting that periodontitis may be associated with structural brain changes. Although these findings do not prove causality, they indicate that periodontal inflammation may contribute to a systemic inflammatory context that favors neuroaxonal damage and progressive neurological dysfunction.

Overall, the included evidence supports a biologically plausible relationship between periodontal disease and multiple sclerosis through shared cytokine networks, NF-κB activation, Th17/Treg imbalance, blood–brain barrier disruption, microglial activation, oral dysbiosis, oxidative stress, MMP activity, complement activation, and neuroaxonal injury. However, most available evidence supports association and mechanistic plausibility rather than direct causality. The proposed bidirectional interaction is summarized in Table 8: periodontal disease may increase systemic inflammatory burden and potentially amplify MS-related neuroinflammation, while MS-related disability, immune dysregulation, xerostomia, autonomic dysfunction, and treatment-related immune modulation may worsen oral hygiene and periodontal inflammatory susceptibility.

### 3.7. Strength of Evidence

The strength of evidence varied according to the methodological quality, directness, and consistency of the contributing studies. Cytokine-mediated inflammation received moderate certainty because it was consistently reported across several sources, although much of the evidence originated from secondary studies with low or moderate methodological confidence. Th17/Treg imbalance, NF-κB signaling, blood–brain barrier dysfunction, and MMP-mediated remodeling were rated low to moderate because they were supported mainly by indirect mechanistic evidence and low-confidence systematic reviews. Pathways supported by fewer studies, substantial heterogeneity, or limited direct evidence in patients with both conditions were assigned low or very low certainty.

Overall, the evidence supports a biologically plausible association between periodontal disease and multiple sclerosis through convergent immunoinflammatory mechanisms. However, no pathway reached a high certainty level, mainly because most available evidence was observational, cross-sectional, mechanistic, or derived from secondary analyses. Therefore, the findings should be interpreted as supportive of association and mechanistic plausibility rather than proof of a direct causal relationship. The results can be seen in Table 9.

### 3.8. Reliability and Validity of Data Extraction

To ensure the reliability and validity of data extraction, a standardized extraction form was used for all included studies. Data extraction was performed independently by three reviewers: A.V.C., A.M.F., and I.R.F. After independent extraction, the results were compared between reviewers, and discrepancies were resolved by majority decision. When necessary, extracted data were rechecked against the full text of the original articles before final inclusion in the synthesis tables. This process was used to reduce subjective interpretation, improve consistency between reviewers, and ensure that the synthesis of findings was based on reproducible and systematically collected data.

Inter-reviewer agreement was assessed using Cohen’s kappa statistic during study selection and data extraction [52]. The number of assessed items ranged from 732 to 733 across reviewer pairs. Agreement between reviewers was high, with Cohen’s kappa values ranging from 0.80 to 0.92, indicating strong to almost perfect agreement. The highest agreement was observed between A.V.C. and A.M.F. (κ = 0.92), while the other reviewer pairs also showed strong agreement. These findings support the reliability and reproducibility of the study selection and data extraction process. The complete inter-reviewer agreement results are presented in Table 10.

## 4. Discussion

The main finding of this systematic review is that periodontal disease and multiple sclerosis appear to share several convergent immunoinflammatory mechanisms, although the available evidence supports biological plausibility rather than direct causality. Periodontal disease and multiple sclerosis are anatomically distinct conditions, affecting the tooth-supporting tissues and the central nervous system, respectively; however, they may be immunologically connected through systemic inflammatory pathways [16,52].

The strongest shared mechanisms identified in this review included cytokine-mediated inflammation [5,7,11,19,20,21,22,23,24,32,45,46,47,48], Th17/Treg imbalance [9,20,21,22,24,45,48], NF-κB activation [19,20,22,23,24,45,46], oxidative stress [7,21,22,23,24,46], matrix metalloproteinase activity [11,20,22,23,24,46], blood–brain barrier dysfunction [19,20,22,23,24,44,45,46,47], oral dysbiosis [22,23,24,45], complement activation [21,23,24,32], and neuroaxonal injury [5,19,21,22,23,44,45,47]. By organizing the synthesis according to inflammatory pathways rather than by individual study type, the present review allowed a more mechanistic interpretation of the available evidence.

Nevertheless, the current literature does not clearly establish whether periodontal disease contributes to multiple sclerosis onset or progression, whether multiple sclerosis increases susceptibility to periodontal disease, or whether both conditions reflect a shared systemic inflammatory phenotype. Therefore, the main unresolved issue is not whether inflammatory overlap exists, but whether this overlap represents cause, consequence, bidirectional interaction, or a common pattern of immune dysregulation.

This interpretation is supported by broader evidence linking periodontitis with systemic diseases such as diabetes [53,54,55], cardiovascular disease [56], rheumatoid arthritis [54,57], kidney disease [57,58], and neurodegenerative disorders [54,59,60,61]. In these conditions, periodontitis is considered a systemic inflammatory amplifier, but the evidence for MS remains less mature, more heterogeneous, and more dependent on indirect mechanistic data.

A relevant contribution of the present review is that it addresses a gap left by previous studies, which have generally examined the relationship between periodontal disease and multiple sclerosis from isolated perspectives [5,11,19,21,22,23,24,32,45,46,47,48]. Much of the available literature has focused on the prevalence of periodontal disease in patients with multiple sclerosis, oral health status, salivary alterations, or systemic inflammation as separate domains. However, few studies have integrated periodontal inflammation, salivary biomarkers, serum inflammatory markers, oral microbiome changes, and neurological outcomes within the same interpretative framework [1,5,11]. In addition, limited attention has been given to differences according to MS phenotype, periodontal disease severity, treatment status, or disability level, all of which may influence the direction and strength of the association.

Another important missing element is the lack of interventional evidence assessing whether periodontal treatment can modify MS-related inflammatory biomarkers or clinical outcomes. Therefore, the value of this review lies in its pathway-based synthesis, which attempts to connect local oral inflammation with systemic and neurological inflammatory mechanisms, rather than simply describing the association between the two diseases [1,11].

Cytokine-mediated inflammation and Th17/Treg imbalance represent two of the most biologically plausible links between periodontal disease and multiple sclerosis, but their causal significance remains uncertain [5,7,11,19,20,21,22,23,24,32,45,46,47,48]. TNF-α, IL-1β, IL-6, and IL-17 are central mediators in both periodontal tissue destruction and MS-related neuroinflammation, suggesting that periodontitis may contribute to systemic inflammatory burden and potentially amplify CNS immune activity [22,23]. Similarly, Th17 polarization may promote both alveolar bone resorption and immune-cell migration across the blood–brain barrier [18,19,20,21]. However, current evidence does not clearly show whether these pathways are initiated by periodontal disease, driven by MS-related immune dysregulation, or reflect a shared pro-inflammatory host phenotype. Longitudinal studies, standardized biomarker panels, and interventional trials assessing cytokine and Th17/Treg changes before and after periodontal treatment in MS patients are still lacking. Therefore, periodontal therapy may be considered a potential strategy to reduce systemic inflammatory load, but it cannot yet be interpreted as an MS-modifying treatment.

It should be noted that cytokines such as IL-1β, IL-6, TNF-α, and IL-17 are not specific to either periodontal disease or multiple sclerosis and are broadly involved in chronic inflammatory responses. Therefore, their shared elevation should be interpreted as evidence of overlapping inflammatory mechanisms rather than a disease-specific biological connection.

Blood–brain barrier dysfunction and microglial activation are central mechanisms in MS, but the contribution of periodontal disease to these pathways remains indirect [19,20,22,23,24,44,45,46,47]. Periodontal cytokines, endotoxins, and MMPs may theoretically weaken endothelial integrity and amplify systemic inflammatory signals that prime microglia [20,22,23,24,46]; however, direct evidence linking periodontal severity with BBB permeability, TSPO-PET findings, smoldering lesions, or brain atrophy in MS is still lacking. Therefore, periodontal disease should be viewed as a possible peripheral amplifier of neuroinflammation, not as an isolated cause of BBB disruption or microglial activation, and periodontal control may only be considered an adjunctive strategy for reducing systemic inflammatory burden.

Salivary biomarkers [1,11] are clinically attractive because they are non-invasive and may reflect oral, systemic, or neurological inflammatory activity. However, their interpretation remains limited by poor standardization and insufficient validation in MS cohorts, so they should currently be considered research tools rather than reliable markers for clinical MS monitoring. Saliva may serve as a translational interface linking oral, systemic, and neuroimmune inflammation rather than merely a source of isolated biomarkers. However, its clinical utility in multiple sclerosis remains investigational and requires standardized sampling protocols, validated biomarker panels, and longitudinal correlation with neurological outcomes.

Importantly, the identified pathways are supported by different levels of evidence. While some mechanisms were consistently reported in human clinical and biomarker studies, others rely mainly on mechanistic or experimental data. Therefore, the findings should be interpreted as supporting biological plausibility and association rather than a direct causal relationship between periodontal disease and multiple sclerosis.

The observed associations may also be influenced by confounding factors such as smoking, oral hygiene, disability level, medication use, vitamin D status, and socioeconomic background. Because many included studies were observational and did not consistently adjust for these variables, the reported relationship between periodontal disease and multiple sclerosis should be interpreted with caution.

The relationship between periodontal disease and multiple sclerosis should be viewed as bidirectional and multifactorial rather than strictly causal. Periodontal disease may increase systemic inflammatory burden, while MS may worsen periodontal status through disability, fatigue, xerostomia, medication effects, and impaired oral hygiene. Shared factors such as smoking, vitamin D deficiency, socioeconomic status, diet, and immune phenotype may further influence both conditions [61,62,63]. MS patients may need closer periodontal monitoring because disability, fatigue, xerostomia, medication effects, and reduced manual dexterity can impair oral hygiene [64,65,66]. Periodontal therapy should not be considered an MS treatment [67], but it may help reduce systemic inflammatory burden as part of broader comorbidity management [68]. Future randomized controlled trials are needed to evaluate this possibility.

This review was strengthened by its PRISMA 2020-guided methodology, OSF registration, predefined eligibility criteria, independent reviewers, inter-reviewer agreement assessment, and risk of bias evaluation using AMSTAR 2, NOS, and JBI tools. In addition, the modified GRADE assessment and pathway-based synthesis allowed a more mechanistic interpretation of the evidence.

Although several pathways were repeatedly reported, their interpretation was limited by the quality of the contributing evidence. Most included systematic reviews had low methodological confidence, while the available primary evidence was limited and largely observational. Therefore, repeated reporting was not interpreted as strong evidence when the underlying studies had important methodological limitations, and conclusions regarding indirect or low-quality pathways were considered preliminary.

The main limitation of the available literature is not the absence of mechanistic plausibility, but the limited directness, standardization, and causal strength of the evidence. Several included studies had low or moderate methodological confidence, and much of the evidence was derived from observational, cross-sectional, or indirect mechanistic data. Additional limitations included heterogeneous periodontal definitions, variable MS phenotypes, inconsistent biomarker panels, incomplete control of confounders, limited longitudinal evidence, absence of interventional periodontal trials in MS patients, and the possibility of publication bias, since the grey literature did not contribute eligible records.

The restriction to studies published between June 2020 and June 2026 may have excluded earlier foundational evidence. Therefore, this review should be interpreted as a synthesis of contemporary findings rather than a comprehensive historical assessment of the periodontal disease–multiple sclerosis relationship.

Including both primary studies and systematic reviews may introduce evidence overlap and overrepresent frequently reported findings. Therefore, certainty was judged according to the quality, directness, and independence of the evidence rather than the number of publications.

Future studies should use prospective MS cohorts with standardized periodontal assessment, MS phenotype stratification, and combined oral, systemic, microbiome, MRI, and neurological biomarkers. Interventional trials are needed to test whether periodontal therapy can reduce systemic inflammation or influence MS-related outcomes.

## 5. Conclusions

This review highlights periodontal disease as more than a localized oral condition in the context of multiple sclerosis, suggesting that it may represent a peripheral inflammatory interface capable of interacting with systemic and neurological immune pathways. Rather than supporting a direct causal model, the available evidence points toward a shared inflammatory environment in which oral dysbiosis, immune activation, and neuroinflammation may influence each other through multiple overlapping mechanisms.

The most important implication of these findings is the need to move beyond a disease-specific view of inflammation. Periodontal disease may contribute to the overall inflammatory burden of patients with multiple sclerosis, while neurological disability and MS-related systemic changes may, in turn, increase periodontal vulnerability. This bidirectional model supports the interpretation of periodontal health as part of broader inflammatory and comorbidity management in MS.

At present, periodontal care should not be considered a disease-modifying treatment for multiple sclerosis. However, maintaining periodontal health may represent a practical and modifiable component of supportive care, with potential benefits for reducing local and systemic inflammatory load. Future studies should determine whether targeted periodontal interventions can influence inflammatory biomarkers, microbiome profiles, or neurological outcomes in well-characterized MS populations.

Overall, the periodontal–multiple sclerosis relationship should be understood as an emerging oral–systemic–neurological inflammatory axis. Its clinical value will depend on future longitudinal and interventional studies capable of clarifying whether periodontal inflammation is only a marker of systemic vulnerability or a modifiable contributor to neuroinflammatory burden.

At present, the relationship between periodontal disease and multiple sclerosis should be interpreted as an emerging hypothesis supported by inflammatory plausibility and shared immunoinflammatory mechanisms but not yet by sufficient longitudinal or interventional evidence to establish directionality or causality.

## Figures and Tables

**Table 1:** Keyword groups used in the literature search.

Domains	Keywords
Multiple sclerosis	“multiple sclerosis”, “MS”, “demyelinating disease”, “neuroinflammatory disease”
Periodontal disease	“periodontal disease”, “periodontitis”, “chronic periodontitis”, “periodontal inflammation”
Oral microbial factors	“oral dysbiosis”, “oral microbiome”, “periodontal pathogens”, “oral bacteria”
Inflammatory mechanisms	“inflammation”, “immune response”, “immune dysregulation”, “systemic inflammation”
Tissue damage pathways	“oxidative stress”, “matrix degradation”, “bone resorption”, “tissue destruction”
Neurological mechanisms	“neuroinflammation”, “blood-brain barrier”, “central nervous system inflammation”, “neurodegeneration”

**Table 2:** Newcastle–Ottawa Scale assessment of observational studies included in the review.

Author, Year	C1	C2	C3	C4	C5	C6	C7	C8	Total
Abdullah, 2025 [19]	1	1	0	0	1	1	1	1	6/9
	Explicit inclusion/exclusion criteria stated	Representative population	No comparator/non-exposed cohort	No control group	Age, sex, EDSS, disease duration	Validated ELISA kits	Same assessment protocol for all patients	12-month follow-up	Moderate risk

C1—Representativeness of the exposed cohort, C2—Selection of the non-exposed cohort, C3—Ascertainment of exposure, C4—Outcome not present at baseline, C5—Comparability of cohorts, C6—Assessment of outcome, C7—Adequate follow-up duration, C8—Adequacy/completeness of follow-up.

**Table 4:** Joanna Briggs Institute critical appraisal assessment of cross-sectional studies included in the review.

Author, Year	C1	C2	C3	C4	C5	C6	C7	C8	Total
Zilberstein et al., 2025 [48]	1	1	1	1	1	1	1	1	8/8
Nie & Lin, 2026 [49]	1	1	1	1	1	1	1	1	8/8

C1—inclusion criteria clearly defined; C2—study subjects and setting described in detail; C3—exposure measured in a valid and reliable way; C4—objective and standard criteria used for measurement of the condition; C5—confounding factors identified; C6—strategies used to deal with confounding factors; C7—outcomes measured in a valid and reliable way; and C8—appropriate statistical analysis used.

**Table 5:** Characteristics and main findings of included studies.

Author, Year & Study Type	Disease Studied	Inflammatory Pathway Names
De Picker et al., 2023 (SR + meta-analysis) [43]	Multiple sclerosis	· Reactive gliosis/microglial activation
		· Astrocyte reactivity
		· TSPO(Translocator Protein)-mediated neuroinflammation
		· CNS-intrinsic innate immune activation
Smusz et al., 2025 (Systematic review) [7]	Multiple sclerosis	· Kynurenine/Indoleamine 2,3-Dioxygenase 1 (IDO-1)/Tryptophan 2,3-Dioxygenase (TDO)pathway
		· IFN-γ/TNF-α cytokine signalling
		· Mitochondrial dysfunction/oxidative stress
		· Arachidonic acid/eicosanoid pathway
		· SCFA/gut–immune axis (TLR signalling)
		· Aryl hydrocarbon receptor (AhR) pathway
		· Nitric oxide/arginine/ADMA pathway (Asymmetric Dimethylarginine)
Abdullah, 2025 (Prospective cohort) [19]	Multiple sclerosis + Periodontal disease	· IL-6/JAK-STAT3 axis
		· TNF-α/NF-κB axis
		· Adhesion molecule upregulation/BBB(Blood–Brain Barrier) transmigration
		· Acute-phase response (IL-6 → CRP(C-Reactive Protein)/ESR(Erythrocyte Sedimentation Rate))
		· Neuroaxonal injury cascade (sNfL)
		· Adaptive vs. innate immune dissociation
Zapata-Acevedo et al., 2022 (Systematic review) [20]	Multiple sclerosis	· MMP-9/MMP-3 proteolytic pathway (NF-κB-driven)
		· IL-1β/TNF-α → NF-κB ECM degradation
		· Laminin-411/integrin α6β1 → Th17 transmigration
		· Laminin-511/tight junction stabilisation
		· COX-2/prostaglandin E2 signalling
		· VEGF-driven angiogenic/BBB destabilisation
		· ECM remodelling feed-forward loop
García-Rios et al., 2025 (Systematic review) [32]	Multiple sclerosis + Periodontal disease	· Complement pathway (CFI)
		· B/T lymphocyte adaptive immune pathway
		· IL-6/TNF-α → RANKL/OPG axis
		· Trained innate immunity/hematopoietic progenitor adaptation
		· Shared gene signatures (FAM46C, CFI, DDIT4L)
		· Autonomic/salivary gland dysfunction pathway
Bagnato et al., 2025 (Systematic review) [44]	Multiple sclerosis	· Smoldering neuroinflammation/chronic innate immune activation
		· Iron-mediated microglial activation
		· TSPO/innate immune cell activation signalling
		· Smoldering demyelination/myelin breakdown
		· Progressive neuroaxonal loss/neurodegeneration cascade
		· BBB-independent chronic inflammation
Gavrilă et al., 2026 (Systematic review) [21]	Multiple sclerosis	· Neuroaxonal injury/NfL release cascade
		· Reactive astrogliosis/GFAP(Glial Fibrillary Acidic Protein) pathway
		· Complement activation (C1q/C3a/C4a/Ba)
		· Complement-GFAP crosstalk (astrocyte-microglia)
		· IL-17/Th17 cytokine axis
		· IL-6/IL-17/IFN-γ cytokine pathway
		· Nitric oxide/nitrosative stress (NOx)
		· Oxidative stress/redox imbalance
		· Cytotoxic B cell/granzyme B pathway
		· CD8+ cytotoxic T cell (GzmB + TEMRA) pathway
		· Gas6/TAM receptor (Tyro3) → white matter degeneration
		· Gut–CNS axis/stool GFAP pathway
		· Choroid plexus expansion/NfL-OPN pathway (Neurofilament Light Chai-Osteopontin)
		· CHI3L1 (YKL-40) Chitinase-3-Like Protein 1/microglial-astrocyte innate immune bridging
Santhanakrishnan et al., 2026 (Systematic review) [22]	Multiple sclerosis + Periodontal disease	· IL-1β/IL-6/TNF-α → BBB tight junction disruption
		· *P. gingivalis*/*T. denticola* LPS → CNS immune activation
		· Oral microbiome dysbiosis → systemic immune dysregulation
		· Oxidative stress (salivary MDA/TOS) → periodontal–CNS axis
		· HPA axis/cortisol dysregulation → oral immune suppression
		· Periodontal inflammation → RANKL/OPG → bone loss and systemic IL-6
		· Oral–immune–neuro axis
Arcas et al., 2024 (saliva) (SR + meta-analysis) [11]	Multiple sclerosis + Periodontal disease	· Salivary TNF-α/IL-1β → systemic and periodontal inflammation
		· Salivary tau proteins/MMPs as CNS-to-oral inflammatory signalling
		· Myelin basic protein (MBP) degradation pathway
		· Acetylcholinesterase (AChE) dysregulation → cholinergic anti-inflammatory pathway
		· Periodontal inflammation → systemic inflammatory burden → MS exacerbation
		· Oral-CNS inflammatory signalling via salivary biomarkers
		· Shared immunological dysregulation (MS-PD bidirectional)
Arcas et al., 2024 (miR-150) (SR + meta-analysis) [5]	Multiple sclerosis	· miR-150/c-Myb axis → T and B cell maturation dysregulation
		· miR-150 → IL-17/IFN-γ/TNF-α cytokine modulation
		· miR-150 upregulation in CSF during MS relapses
		· miR-150-5p downregulation in SPMS → BDNF-mediated neurodegeneration
		· miR-150 in myeloid extracellular vesicles → cognitive impairment
		· miR-150 epigenetic regulation of MS inflammatory pathways
		· miR-150/natalizumab-induced immune cell redistribution
		· miR-150 CIS-to-MS conversion biomarker pathway
Martínez-Martínez et al., 2025 (Systematic review) [47]	Multiple sclerosis + Periodontal disease	· *P. gingivalis* BBB translocation and neuroinflammation
		· Oral dysbiosis-driven systemic LPS-mediated cytokine cascade
		· Amyloid-β accumulation and tau hyperphosphorylation via periodontal LPS
		· Trigeminal nerve as direct oral-brain inflammatory conduit
		· *P. gingivalis* disruption of oral microbiota homeostasis
		· Subgingival dysbiotic microbiome composition shift
		· Oral–gut–brain axis dysregulation → neurofibrillary tangle initiation
Zilberstein et al., 2025 (Cross-sectional) [48]	Multiple sclerosis + Periodontal disease	· Gum–gut axis: oral pathobiont translocation to intestinal mucosa
		· Streptococcus-mediated intestinal mucosal damage and NO dysregulation
		· Granulicatella arginine-citrulline pathway and intestinal permeability
		· Actinomyces PPIase-endopeptidase suppression of innate immunity
		· Tryptophan and arginine-citrulline dysregulation → Th17/Treg imbalance
		· Periodontal inflammation-driven systemic cytokine elevation (CRP, IL-6)
		· Diet–microbiota interaction: reduced Mediterranean diet adherence
Salhi et al., 2023 (Systematic review) [23]	Multiple sclerosis + Periodontal disease	· TLR4/NF-κB signalling activation by Pg-LPS → neuroinflammation
		· Amyloid-β accumulation and APP processing dysregulation
		· Tau hyperphosphorylation via PP2A inhibition → neurofibrillary tangles
		· BBB disruption via claudin-5 reduction and periodontal IL-6
		· Gingipain-mediated microglial activation via PAR2/PI3K/ERK
		· CatB/NF-κB pathway and RAGE upregulation → amyloid accumulation
		· Complement system activation by *P. gingivalis* → chronic glial inflammation
		· STAT3 signalling pathway activation by periodontitis-induced LPS
		· Metastatic brain infection by periodontal pathogens
Dhungana et al., 2025 (Systematic review) [24]	Periodontal disease	· TLR2/4-NF-κB-mediated endothelial activation and cytokine release
		· ROS/NO dysregulation via eNOS/iNOS/Nrf2/BH4 axis
		· Gingipain-mediated ApoB-100 degradation/foam cell formation
		· *F. nucleatum* FadA/GroEL-mediated endothelial permeability (TLR-MyD88-NF-κB)
		· *T. forsythia* BspA-mediated LXRα/LXRβ/ABCA1 downregulation
		· *T. denticola* dentilisin-mediated cytokine degradation and vascular damage
		· MMP/TIMP dysregulation/fibrinogen elevation/plaque destabilisation
		· Th17/Treg imbalance and anti-Pg antibody cardiovascular cross-reactivity
Li et al., 2026 (SR + meta-analysis) [46]	Periodontal disease	· Periodontal bacteremia and systemic pathogen dissemination
		· IL-6 and TNF-α pro-inflammatory cytokine elevation
		· hs-CRP acute-phase response and cardiovascular risk amplification
		· Endothelial dysfunction via periodontal pathogen-driven inflammation
		· Platelet aggregation and thrombosis formation
		· Atherosclerotic plaque destabilisation and rupture
		· NF-κB signalling activation by periodontal pathogens
		· Cumulative periodontal bone destruction as chronic systemic inflammatory burden
Nie & Lin, 2026 (Cross-sectional) [49]	Periodontal disease	· Oral microbial dysbiosis—reduced diversity enabling pathogen dominance
		· Fusobacterium and Prevotella expansion driving local and systemic inflammation
		· Loss of protective commensals (Streptococcus, Actinomyces) and immune dysregulation
		· Biofilm-mediated gingival inflammation and epithelial barrier disruption
		· Oral microbiome composition as predictor of systemic inflammatory burden
Arriaga et al., 2026 (Systematic review) [45]	Periodontal disease	· TNF-α elevation linking periodontal infection to AD neurodegeneration
		· Periodontal pathogen IgG antibodies as markers of CNS inflammatory burden
		· Blood–brain barrier penetration by periodontal endotoxins
		· IL-6 and IL-1β mediated neuroinflammatory amplification and Aβ/tau cascade
		· Chronic periodontal infection and cognitive decline via APOE-ε4 modulation
		· Periodontal pathogen-driven neuroinflammation → MRI-detectable neurodegeneration
		· Trigeminal nerve pathway for periodontal cytokine CNS access

**Table 6:** Shared immunoinflammatory pathways between periodontal disease and multiple sclerosis.

Shared Pathway	Role in Periodontal Disease	Role in Multiple Sclerosis
TNF-α/NF-κB axis [5,7,11,19,20,22,23,24,32,45,46,47,48]	Released from periodontal tissues; drives gingival destruction, bone resorption, and systemic inflammation	Activates microglial and astrocyte reactivity; promotes BBB disruption and smoldering neuroinflammation
IL-6/JAK-STAT3 pathway [19,22,23,24,32,45,46,48]	Drives RANKL/OPG imbalance causing alveolar bone loss; elevates CRP and acute-phase proteins systemically	Promotes Th17 over Treg differentiation; upregulates adhesion molecules on BBB endothelium
IL-1β innate immune activation [2,3,6,9,14,15,16,19,21,25,26,29]	Secreted by gingival macrophages; amplifies local tissue destruction and systemic cytokine cascade	Produced by activated microglia; promotes demyelination, neuroaxonal injury, and Aβ synthesis
TLR2/4 → NF-κB via microbial LPS [22,23,24,45,46,47,48]	Periodontal Gram-negative LPS activates TLR2/4 → NF-κB → IL-1β, TNF-α, MMP release, endothelial dysfunction	Periodontal LPS crossing the BBB activates TLR4/NF-κB in microglia → M1 polarisation and demyelination
Blood–brain barrier disruption [19,20,22,23,24,44,45,46,47]	*P. gingivalis* gingipains reduce claudin-5 expression; IL-6 increases endothelial permeability	MMP-9/MMP-3 degrade tight junction proteins; laminin-411 isoform switch facilitates Th17 CNS infiltration
Th17/Treg imbalance [19,20,21,22,48]	Chronic periodontal infection skews immunity toward Th17; anti-Pg antibodies cross-react with host proteins	Th17 cells are central MS effectors; IL-17 drives BBB transmigration and CNS demyelination
Complement activation [21,23,24,32]	*P. gingivalis* gingipains cleave C3/C5 generating anaphylatoxins; CFI gene dysregulated in PD	C1q, C3a, C4a elevated in MS lesions; complement-GFAP astrocyte-microglia crosstalk perpetuates gliosis
Oxidative stress/ROS/NO dysregulation [7,21,22,23,24,46]	Neutrophil ROS and eNOS/iNOS/Nrf2/BH4 dysregulation drive endothelial damage and periodontal destruction	Mitochondrial ROS and NOx overproduction promote oligodendrocyte apoptosis and axonal injury in MS lesions
MMP-mediated tissue remodelling [11,20,22,23,24,46]	MMP-8/MMP-9 degrade collagen and alveolar bone matrix; systemic MMP elevation damages vascular endothelium	MMP-9/MMP-3 degrade BBB tight junctions enabling leukocyte transmigration and active demyelination
Oral microbiome dysbiosis [22,24,45,47,48,49]	Reduced microbial diversity; Fusobacterium/Prevotella expansion; Streptococcus/Actinomyces depletion	Oral dysbiosis drives LPS translocation and oral–gut–brain axis disruption amplifying CNS neuroinflammation
Oral–gut–brain axis [7,23,47,48,49]	Periodontal pathobionts translocate to intestinal mucosa, increasing gut permeability and pro-inflammatory metabolites	Gut dysbiosis contributes to MS via Short-Chain Fatty Acid (SCFAs), tryptophan/kynurenine metabolism, and Th17/Treg imbalance
Kynurenine/IDO-1/tryptophan pathway [7,22,48]	Periodontal IFN-γ activates IDO-1; disrupted tryptophan and arginine-citrulline profiles documented in PD/MS patients	IDO-1/TDO overactivation diverts tryptophan toward neurotoxic quinolinic acid, depleting neuroprotective serotonin
Trigeminal nerve inflammatory conduit [47]	TNF-α, IL-1β, IL-6 from periodontal tissues travel via cranial nerve V toward the CNS trigeminal nucleus	Direct oral-to-CNS conduit bypassing BBB; may initiate or amplify neuroinflammatory cascades in brainstem lesions
Microglial activation (TSPO) [22,23,43,44]	Periodontal LPS activates microglia via TLR4 and PAR2/PI3K/ERK upon CNS entry; promotes M1 polarisation	TSPO-positive smoldering microglial activation drives progressive neuroaxonal loss between relapses in MS
Neuroaxonal injury (NfL/GFAP) [5,19,21,22,44,45]	Active periodontitis correlates with higher sNfL in MS patients, linking periodontal inflammation to axonal damage	sNfL and GFAP are validated MS neuroaxonal injury biomarkers; elevated across all subtypes during active disease
Shared gene signatures [5,32,49]	FAM46C, CFI, DDIT4L dysregulated in PD; complement and metabolic stress gene overlap with MS populations	Same gene signatures (CFI, FAM46C, DDIT4L) dysregulated in MS; epigenetic overlap via miR-150 regulation

**Table 7:** Inflammatory evidence across periodontal, salivary, systemic, microbial, and neurological compartments.

Compartment	Markers/Mechanisms	Relevance to Periodontal Disease	Relevance to MS	Evidence Strength
Periodontal [11,22,24,32,45,46,48,49]	Biofilm, periodontal pathogens, IL-1β, IL-6, TNF-α, IL-17, MMPs, RANKL	Dysbiotic biofilm drives gingival tissue destruction, CAL, alveolar bone loss, and pocket formation via MMP/RANKL activation	Periodontal severity (CAL, BOP) independently correlates with MS disability and sNfL elevation	High
Salivary [5,11,22,48,49]	Cytokines, oxidative stress markers, MMPs, tau, MBP, miRNAs	Elevated salivary TNF-α, IL-1β, MMPs, and oxidative stress markers (MDA, TOS) reflect active periodontal inflammation	Salivary tau, MBP degradation products, and miR-150 serve as CNS-to-oral inflammatory signalling markers in MS	Moderate
Serum/systemic [11,19,22,23,24,45,46]	CRP, fibrinogen, cytokines, activated leukocytes, oxidative stress, IgG antibodies	Periodontal bacteremia elevates systemic CRP, IL-6, TNF-α, and fibrinogen independently of cardiovascular risk factors	Elevated serum NfL, GFAP, TNF-α, and IgG antibodies to periodontal pathogens correlate with MS neurodegeneration and cognitive decline	Moderate
Microbial [22,23,24,45,47,48,49]	Oral dysbiosis, *Porphyromonas gingivalis*, LPS, PAMPs, gingipains, *T. denticola*, *F. nucleatum*	Subgingival dysbiosis dominated by Gram-negative anaerobes produces LPS, gingipains, and PAMPs that activate TLR2/4 → NF-κB cascade	Periodontal pathogens detected in brain tissue; *P. gingivalis* gingipains activate microglia and promote Aβ accumulation and tau hyperphosphorylation	High
Neurological/CNS [5,19,20,21,22,23,43,44,45,47]	BBB disruption, microglial activation, astrocytic activation, NfL, GFAP, demyelination	Periodontal LPS crosses BBB via gingipain-mediated claudin-5 reduction; correlated with MRI-detectable hippocampal atrophy and white matter hyperintensities	TSPO-positive smoldering microglial activation, reactive astrogliosis (GFAP), and progressive neuroaxonal loss (sNfL) are hallmarks of MS neurodegeneration	Moderate

**Table 8:** Proposed bidirectional interactions between periodontal disease and multiple sclerosis.

Direction	Proposed Mechanism	Biological Consequence
Periodontal disease → Multiple sclerosis [11,22,23,24,32,45,46,47,48]	Periodontal pathogens, LPS, gingipains, PAMPs	Systemic bacteremia and endotoxaemia activate TLR/NF-κB → pro-inflammatory cytokines; pathogens cross BBB and activate microglia
	IL-1β, IL-6, TNF-α systemic cytokine spillover	May increase systemic immune activation, BBB permeability, and smoldering neuroinflammation in MS
	Oxidative stress and MMP activation	May contribute to BBB tight junction degradation, tissue injury, and inflammatory amplification in CNS lesions
	Oral–gut–brain axis dysbiosis and tryptophan/IDO-1 pathway disruption	Oral pathobiont translocation to gut alters microbiota composition, amplifying Th17/Treg imbalance and neuroinflammation
	Trigeminal nerve direct inflammatory conduit	Periodontal cytokines and neuropeptides travel via cranial nerve V to CNS, bypassing BBB and amplifying brainstem neuroinflammation
Multiple sclerosis → Periodontal disease [11,19,21,22,32]	Disability, fatigue, and reduced manual dexterity	Impaired ability to perform oral hygiene increases plaque accumulation, gingival inflammation, and periodontal disease risk
	Autonomic dysfunction and xerostomia	Reduced salivary flow impairs oral mucosal defense, promotes dysbiosis, and increases susceptibility to periodontal pathogens
	HPA axis dysregulation and cortisol elevation	Chronic stress-driven cortisol suppresses local oral immune responses, reducing neutrophil effectiveness against periodontal biofilm
	CNS-driven systemic immune dysregulation	MS-associated Th17 dominance and reduced Treg function may amplify host periodontal inflammatory response to dysbiotic biofilm
MS therapy → oral inflammation [5,22,32]	Immunomodulatory drugs (natalizumab, interferons, glatiramer)	Immune modulation alters host response to periodontal biofilm; may reduce systemic cytokine amplification of periodontal inflammation
	Corticosteroid use and immunosuppression	Long-term corticosteroid or immunosuppressive therapy may mask periodontal inflammatory signs and impair local tissue repair

**Table 9:** Certainty of evidence for shared immunoinflammatory pathways between periodontal disease and multiple sclerosis using a modified GRADE approach.

Inflammatory Pathway	Supporting Evidence	Main Limitations	Certainty of Evidence
Cytokine-mediated systemic inflammation	Recurrent evidence for TNF-α, IL-1β, IL-6, and IL-17 involvement in periodontal inflammation, systemic immune activation, and MS-related neuroinflammation.	Heterogeneity in biomarker assessment and limited longitudinal evidence directly linking periodontal inflammation to MS progression.	Moderate
Th17/Treg imbalance	Supported by evidence from both periodontal disease and MS, with IL-17 involved in periodontal bone loss, BBB dysfunction, and autoimmune neuroinflammation.	Mostly mechanistic or indirect evidence; limited studies assessing Th17/Treg balance in patients with both conditions.	Moderate
NF-κB signaling	Identified as a common molecular pathway activated by periodontal pathogens and involved in cytokine release, endothelial activation, and MMP expression.	Evidence partly extrapolated from systemic inflammatory and neurodegenerative models.	Low to moderate
Blood–brain barrier dysfunction	Supported by evidence linking cytokines, MMPs, endothelial activation, and periodontal endotoxins with BBB permeability.	Limited direct clinical evidence demonstrating periodontal-driven BBB disruption in MS patients.	Low to moderate
MMP-mediated extracellular matrix remodeling	MMPs are involved in periodontal connective tissue destruction and in BBB/extracellular matrix disruption in MS.	Variability in MMP measurement and limited direct comparative data.	Low to moderate
Oxidative and metabolic stress	Oxidative stress contributes to periodontal tissue injury and MS-related mitochondrial dysfunction, demyelination, and neurodegeneration.	Heterogeneous oxidative stress markers and limited disease-specific validation.	Low
Oral dysbiosis and oral–gut–brain axis	Oral microbiome alterations, periodontal pathogens, and tryptophan/kynurenine metabolism may link oral inflammation with systemic and CNS immune activation.	Emerging evidence; limited longitudinal and interventional studies.	Low
Complement activation	Complement dysregulation appears relevant in both periodontal innate immune responses and MS-related glial activation.	Supported by fewer studies and mainly mechanistic evidence.	Low
miRNA and epigenetic regulation	miR-150 and shared gene signatures may connect immune regulation, neuroinflammation, and periodontal susceptibility.	Limited number of studies and insufficient direct validation in combined MS–periodontitis cohorts.	Very low to low
Salivary biomarkers	Salivary cytokines, MMPs, oxidative stress markers, and neurological biomarkers may reflect oral–systemic inflammatory communication.	Promising but heterogeneous; limited diagnostic standardization and clinical validation.	Low
Neuroaxonal injury and structural CNS damage	NfL, GFAP, microglial activation, and structural CNS changes may represent downstream consequences of sustained systemic inflammation.	Association supported, but causality between periodontal disease and neuroaxonal injury remains unproven.	Low to moderate

**Table 10:** Reliability and validity procedures applied during data extraction.

Reviewer Pair	Number of Assessed Items	Agreed Items	Disagreed Items	Cohen’s Kappa Value	Interpretation
A.V.C. & A.M.F.	732	704	28	0.92	Almost perfect agreement
A.V.C. & I.R.F.	732	660	72	0.80	Strong agreement
A.M.F. & I.R.F.	733	678	55	0.85	Strong agreement

## Data Availability

No new datasets were generated during this systematic review. The data supporting the reported results are derived from previously published studies included in this review and are available in the respective original publications. The protocol of this systematic review was registered on the Open Science Framework (OSF) under the registration code osf.io/qmex2 to ensure methodological transparency and reproducibility and to reduce the risk of selective reporting. All extracted data, including the list of excluded articles, were uploaded to the OSF registry associated with this registration, under the registration DOI 10.17605/OSF.IO/K4DU.

## Abbreviations

The following abbreviations are used in this manuscript:

B-cell	B lymphocyte/B cell
Th1	T helper 1 cell subset
Th17	T helper 17 cell subset
IL-17	Interleukin-17
IL-6	Interleukin-6
IL-1β	Interleukin-1 beta
TNF-α	Tumor necrosis factor alpha
IFN-γ	Interferon gamma
GM-CSF	Granulocyte-macrophage colony-stimulating factor
RANKL	Receptor activator of nuclear factor kappa-B ligand
OPG	Osteoprotegerin
CNS	Central nervous system
MS	Multiple sclerosis
PRISMA	Preferred Reporting Items for Systematic Reviews and Meta-Analyses
MEDLINE	Medical Literature Analysis and Retrieval System Online
AMSTAR 2	A Measurement Tool to Assess Systematic Reviews 2
GRADE	Grading of Recommendations, Assessment, Development and Evaluations
OSF	Open Science Framework
DOI	Digital Object Identifier
URL	Uniform Resource Locator
miR-150	microRNA-150
PICO	Population, Intervention, Comparator, Outcome
JBI	Joanna Briggs Institute
16S rRNA	16S ribosomal ribonucleic acid
WHO	World Health Organization
NOS	Newcastle–Ottawa Scale
NF-κB	Nuclear factor kappa-light-chain-enhancer of activated B cells
TLR2	Toll-like receptor 2
TLR4	Toll-like receptor 4
MMP-3	Matrix metalloproteinase-3
MMP-9	Matrix metalloproteinase-9
ZO-1	Zonula occludens-1
MMP	Matrix metalloproteinase
Treg	Regulatory T cell
TSPO-PET	Translocator protein positron emission tomography
LPS	Lipopolysaccharide
NfL	Neurofilament light chain
GFAP	Glial fibrillary acidic protein
sNfL	Serum neurofilament light chain
BBB	Blood–brain barrier
MRI	Magnetic resonance imaging
IL-23	Interleukin-23
ROBIS	Risk of Bias in Systematic Reviews
EDSS	Expanded Disability Status Scale
ELISA	Enzyme-linked immunosorbent assay
SR	Systematic review
IDO-1	Indoleamine 2,3-dioxygenase 1
TDO	Tryptophan 2,3-dioxygenase
SCFA	Short-chain fatty acid
TLR	Toll-like receptor
AhR	Aryl hydrocarbon receptor
ADMA	Asymmetric dimethylarginine
JAK-STAT3	Janus kinase/signal transducer and activator of transcription 3 pathway
CRP	C-reactive protein
ESR	Erythrocyte sedimentation rate
ECM	Extracellular matrix
COX-2	Cyclooxygenase-2
VEGF	Vascular endothelial growth factor
CFI	Complement factor I
FAM46C	Family with sequence similarity 46 member C
DDIT4L	DNA damage-inducible transcript 4-like
C1q/C3a/C4a/Ba	Complement components/fragments C1q, C3a, C4a and Ba
CD8+	Cluster of differentiation 8 positive T cells
GzmB + TEMRA	Granzyme B-positive terminally differentiated effector memory T cells re-expressing CD45RA
Gas6	Growth arrest-specific 6
TAM	Tyro3, Axl and MerTK receptor family
Tyro3	Tyrosine-protein kinase receptor Tyro3
CHI3L1	Chitinase-3-like protein 1
YKL-40	Chitinase-3-like protein 1 biomarker, also known as YKL-40
MDA	Malondialdehyde
TOS	Total oxidative status
HPA	Hypothalamic–pituitary–adrenal axis
MBP	Myelin basic protein
AChE	Acetylcholinesterase
CSF	Cerebrospinal fluid
SPMS	Secondary progressive multiple sclerosis
BDNF	Brain-derived neurotrophic factor
CIS	Clinically isolated syndrome
NO	Nitric oxide
Pg-LPS	*Porphyromonas gingivalis* lipopolysaccharide
APP	Amyloid precursor protein
PP2A	Protein phosphatase 2A
PAR2	Protease-activated receptor 2
PI3K	Phosphoinositide 3-kinase
ERK	Extracellular signal-regulated kinase
CatB	Cathepsin B
RAGE	Receptor for advanced glycation end-products
STAT3	Signal transducer and activator of transcription 3
ROS	Reactive oxygen species
eNOS	Endothelial nitric oxide synthase
iNOS	Inducible nitric oxide synthase
Nrf2	Nuclear factor erythroid 2-related factor 2
BH4	Tetrahydrobiopterin
ApoB-100	Apolipoprotein B-100
FadA	Fusobacterium adhesin A
MyD88	Myeloid differentiation primary response 88
LXRα/LXRβ/ABCA1	Liver X receptors alpha/beta and ATP-binding cassette transporter A1
TIMP	Tissue inhibitor of metalloproteinases
hs-CRP	High-sensitivity C-reactive protein
AD	Alzheimer’s disease
IgG	Immunoglobulin G
APOE-ε4	Apolipoprotein E epsilon 4 allele
M1	Classically activated pro-inflammatory macrophage/microglial phenotype
PD	Periodontal disease
MMP-8	Matrix metalloproteinase-8
SCFAs	Short-chain fatty acids
CAL	Clinical attachment loss
BOP	Bleeding on probing
PAMPs	Pathogen-associated molecular patterns
